# Self-Assembly with 2,6-Bis(1-(pyridin-4-ylmethyl)-1*H*-1,2,3-triazol-4-yl)pyridine: Silver(I) and Iron(II) Complexes

**DOI:** 10.3390/molecules22101762

**Published:** 2017-10-19

**Authors:** Daniel A. W. Ross, Dan Preston, James D. Crowley

**Affiliations:** Department of Chemistry, University of Otago, P.O. Box 56, Dunedin 9016, Otago, New Zealand; rosda272@student.otago.ac.nz

**Keywords:** silver(I), CuAAC “click”, iron(II), metallosupramolecular, co-ordination polymer, self-assembly

## Abstract

A new “click” ligand, 2,6-bis(1-(pyridin-4-ylmethyl)-1*H*-1,2,3-triazol-4-yl)pyridine (**L**) featuring a tridentate 2,6-bis(1,2,3-triazol-4-yl)pyridine (tripy) pocket and two pyridyl (py) units was synthesized in modest yield (42%) using the copper(I) catalyzed azide-alkyne cycloaddition (CuAAC) reaction. The coordination chemistry of the ligand with silver(I) and iron(II) ions was examined using a battery of solution (^1^H and DOSY (diffusion ordered spectroscopy) nuclear magnetic resonance (NMR), infrared and absorption spectroscopies, high-resolution electrospray ionization mass spectrometry (HR-ESI-MS)), and solid state (X-ray crystallography, elemental analysis) techniques. When treated with silver(I) ions, the ligand forms discrete [Ag(**L**)]^+^ (X^−^, where X^−^ = BF_4_^−^, NO_3_^−^ or SbF_6_^−^) complexes in dimethyl sulfoxide (DMSO) solution but these complexes crystallize as coordination polymers. The addition of [Fe(H_2_O)_6_](BF_4_)_2_ to an acetonitrile solution of the ligand forms the expected monomeric octahedral [Fe(**L**)_2_]^2+^ complex and treatment of the iron(II) complex with AgBF_4_ generates a heterometallic linear coordination polymer.

## 1. Introduction

The generation of self-assembled metallosupramolecular architectures has been studied extensively in the past 30 years [1]. The main factors controlling the outcome of the self-assembly process are now reasonably well understood, appropriate matching of the coordination geometry preference of the metal ion(s), the denticity of the ligands, and the length and flexibility of the linking units between binding sites on the ligands usually provides good-to-excellent control on the resulting assembly. The combination of a suitable metal ion with a rigid bridging ligand will reliably generate either discrete macrocyclic or cage architectures or continuous metal-organic framework materials. However, bridging ligands with more flexibility provide less control on the outcome of the self-assembly process and can lead to mixtures or coordination polymers [2]. Coordination polymers can also be generated by exploiting rigid ligands with divergent coordinating units. As these metallosupramolecular systems can be readily self-assembled, as opposed to generated through (potentially) more laborious step-by-step synthetic procedures, they have been examined for a wide range of applications, including gas sorption [3,4], drug delivery [5,6], and catalysis [7,8]. Metallosupramolecular systems with interesting biological [9], photophysical [10,11] and redox [12,13,14,15] properties have also been generated. Recently, as a part of efforts to further widen the potential applications of metallosupramolecular architectures, there has been a focus on the design and synthesis of heterometallic [16,17] complexes/assemblies.

**Figure 1:**
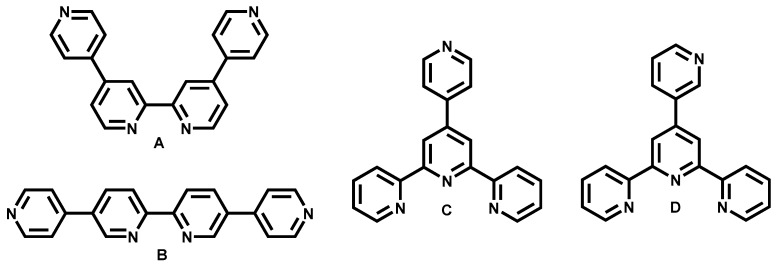
2,2-Bipyridine (bipy) and 2,2′:6′,2″-terpyridine (terpy) chelators conjugated to pyridyl units, 4,4′:2′,2″:4″,4″′-quaterpyridine (**A**), 4,5′:2′,2″:5″,4″′-quaterpyridine (**B**), 4′-(4-pyridinyl)-2,2′:6′,2″-terpyridine (**C**) and 4′-(3-pyridinyl)-2,2′:6′,2″-terpyridine (**D**).

There are potentially several different approaches to the generation of heterometallic architectures and ligands that feature binding pockets with different denticities (e.g., bidentate and monodentate or tridentate and monodentate) have been used to synthesize both discrete and polymeric systems. In particular, classical 2,2-bipyridine (bipy) and 2,2′:6′,2″-terpyridine (terpy) chelators conjugated to pyridyl units have received considerable attention (Figure 1A–D). The chelate effect leads to the initial complexation of a metal ion within the bidentate or tridentate pocket of the ligands, while the addition of a second equivalent of the same or a different metal ion generates either discrete [18,19,20,21] or polymeric [22,23,24,25,26,27,28] metallosupramolecular assemblies.

Recently, the 2-(1-**R**-1*H*-1,2,3-triazol-4-yl)pyridine (R-pytri) and 2,6-bis(1-**R**-1*H*-1,2,3-triazol-4-yl)pyridine (R-tripy) chelator units have emerged as alternatives to the more well established bipy and terpy ligands. The interest in the R-pytri and R-tripy ligands is fueled, in part, by the fact that the compounds are synthesized using the mild functional group tolerant copper(I) catalyzed alkyne-azide 1,3-cycloaddition (CuAAC) “click” reaction. This enables a wide variety of functionalized “click” ligands to be generated for different applications. We and others have used “click” ligands to assemble a variety of discrete metallosupramolecular architectures. [29] Herein, we build on those studies and report the synthesis of the new “click” ligand, 2,6-bis(1-(pyridin-4-ylmethyl)-1*H*-1,2,3-triazol-4-yl)pyridine (**L**). The ligand features a central tridentate 2,6-bis-(1*H*-1,2,3-triazol-4-yl)pyridine (tripy) binding pocket and two peripheral pyridyl units. The self-assembled metallosupramolecular architectures generated from **L** and silver(I) and iron(II) ions are examined using solution and solid state techniques.

## 2. Results and Discussion

### 2.1. Ligand Synthesis and Homometallic Complexation

**Scheme 1:**
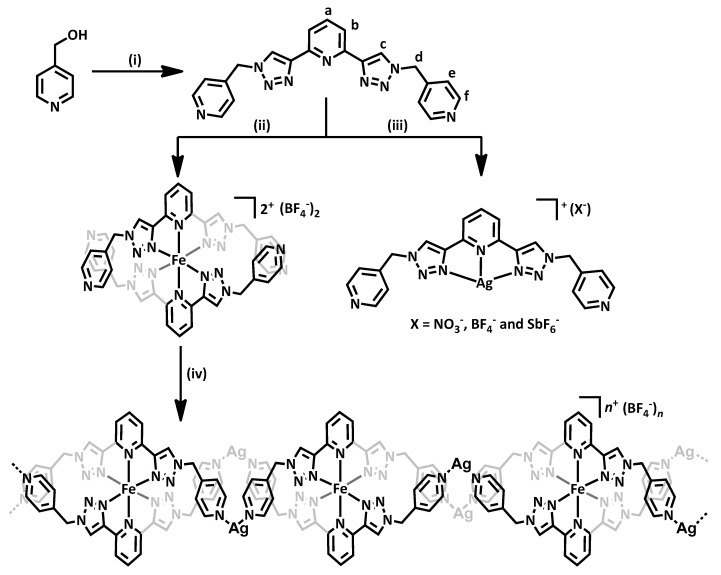
Synthesis of the ligand (**L**) and the corresponding silver(I) and iron(II) complexes: (**i**) (**a**) MsCl, NEt_3_, DMF, 1 h; (**b**) NaN_3_, DMF, RT, 20 h, (**c**) 2,6-diethynylpyridine [30], CuSO_4_·5H_2_O, sodium ascorbate, DMF/H_2_O (4:1), RT, 20 h; (**ii**) [Fe(OH_2_)_6_](BF_4_)_2_, CH_3_CN, RT, 5 min; and, (**iii**) AgX (where X = BF_4_^−^, NO_3_^−^ or SbF_6_^−^), DMF, RT, 5 min (**iv**) AgBF_4_, CH_3_CN/THF, RT, 24 h.

The “click” ligand, 2,6-bis(1-(pyridin-4-ylmethyl)-1*H*-1,2,3-triazol-4-yl)pyridine (**L**) was synthesized from 4-pyridinemethanol and 2,6-diethynylpyridine [30] without the need to isolate the intermediate azide (Scheme 1). 4-Pyridinemethanol (3 equiv.) and methanesulfonyl chloride (MsCl, 3 equiv.) were stirred in dimethylformamide (DMF) with triethylamine (NEt_3_) for 1 h to generate the methanesulfonate ester of 4-pyridinemethanol [31]. Sodium azide (2.5 equiv.) was then added to the reaction mixture and the resulting solution was stirred at room temperature overnight (20 h). The DMF solution of the in situ generated 4-(azidomethyl)pyridine was then added to a mixture of 2,6-diethynylpyridine (1 equiv.), CuSO_4_·5H_2_O and sodium ascorbate in DMF/H_2_O (4:1) and stirred at room temperature for 20 h (Scheme 1) providing, after work up, the desired ligand in modest yield (42%).

The new ligand, **L**, was characterised via ^1^H-NMR, ^13^C-NMR, and infrared spectroscopies and high-resolution electrospray mass spectrometry (HR-ESI-MS) and elemental analysis (Scheme 1, Supplementary Materials, Figures S1 and S2).

### 2.2. Homometallic Silver(I) and Iron(II) Complexes

Diamagnetic, labile, d^10^ silver(I) ions have been used extensively in the construction of metallosupramolecular architectures because they can adopt a wide variety of coordination numbers (ranging from 2 to 6) and geometries. We have previously shown that silver(I) forms interesting polynuclear complexes with bi- and tri-dentate pyridyl-1,2,3-triazole ligands [32,33,34] and metallomacrocycles [35] with pyridyl based ligands, thus we initially examined the complexation of 2,6-bis(1-(pyridin-4-ylmethyl)-1*H*-1,2,3-triazol-4-yl)pyridine (**L**) with a range of silver(I) salts (Scheme 1).

The silver(I) complexes of **L** were prepared by adding an acetonitrile or acetone solution of AgX (1 equiv., where X = BF_4_^−^, NO_3_^−^ or SbF_6_^−^) to an acetonitrile or acetone solution of the ligand (**L** 1 equiv.). The resulting solutions were stirred at room temperature for 5 min in the absence of light (Scheme 1) and colorless solids immediately precipitated in each case. Repeating the synthesis in dimethylformamide (DMF), resulted in colorless solutions. Vapor diffusion of diethyl ether into the DMF solutions generated either X-ray quality colorless crystals or microcrystalline powders in good yields (50–92%). IR spectra of the isolated colorless solids show absorption bands resulting from C-H stretching (3100–2900 cm^−1^) and from the skeletal vibrations of the aromatic rings (1600–1400 cm^−1^) confirming the presence of the ligands in the isolated materials, while elemental analysis indicated that complexes with 1:1 metal:ligand ratios were obtained.

**Figure 2:**
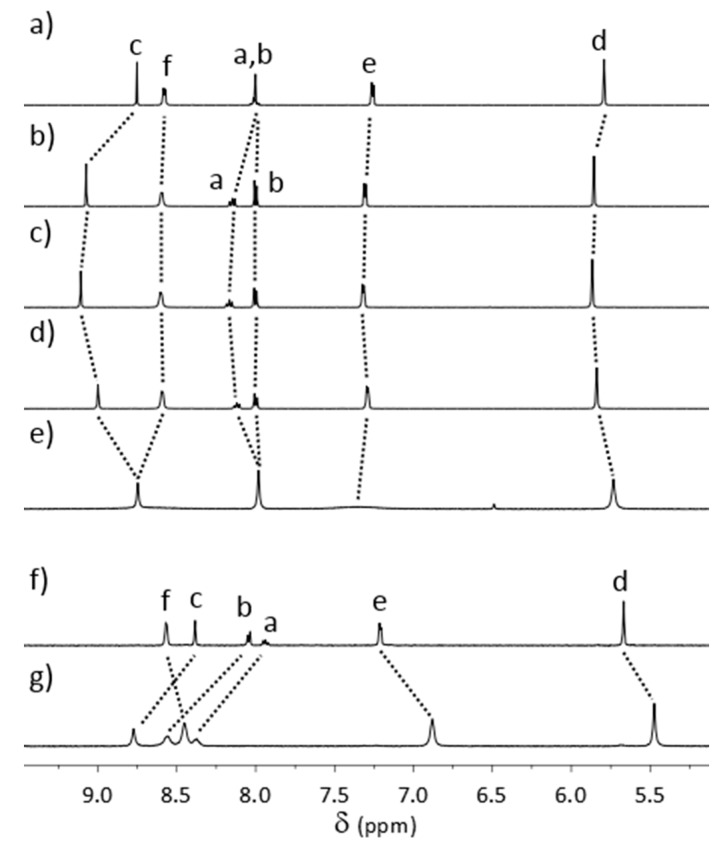
Partial stacked ^1^H-NMR spectra (500 MHz, *d*_6_-DMSO, 298 K) of (**a**) **L**; (**b**) [Ag(**L**)]NO_3_; (**c**) [Ag(**L**)]BF_4_; (**d**) [Ag(**L**)]SbF_6_ and (**e**) [Fe(**L**)_2_](BF_4_)_2_ and (500 MHz, *d*_3_-acetonitrile, 298 K) (**f**) **L** and (**g**) [Fe(**L**)_2_](BF_4_)_2_.

**Figure 3:**
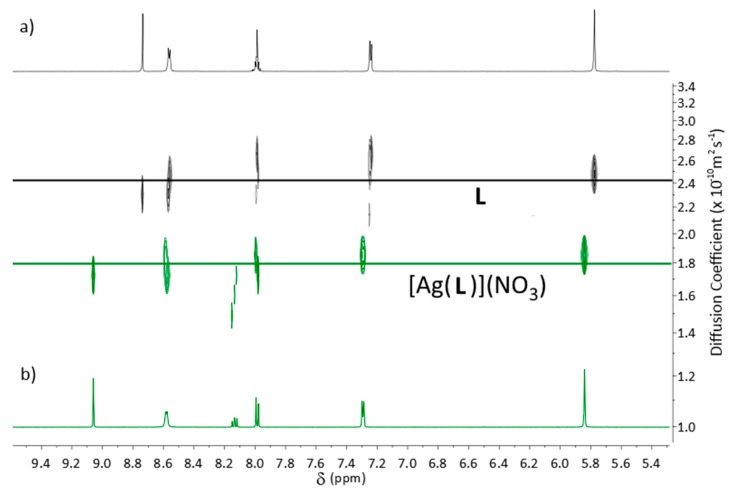
Partial stacked ^1^H DOSY NMR spectra (500 MHz, *d*_6_-DMSO, 298 K) of (**a**) **L** (black trace), (**b**) [Ag(**L**)]NO_3_ (green trace).

The complexes were only soluble in polar DMF or DMSO, ^1^H NMR spectroscopy (500 MHz, *d*_6_-DMSO, 298 K) of the three complexes showed the downfield shifting of ligand protons H_a_ (Δδ = 0.12–0.17 ppm) and H_c_ (Δδ = 0.25–0.32 ppm), relative to the “free” ligand consistent with complex formation (Figure 2, Supplementary Materials, Figures S3–S9). No/negligible shifts of the H_e_ and H_f_ protons of the pendent pyridyl group suggested that the silver(I) ions are coordinated in the central tripy tridentate pocket only, and are not interacting with the pendent pyridines in solution. ^1^H DOSY experiments (Table 1, Figure 3) confirmed that for the three silver compounds, a 1:1 complex of the ligand and silver forms in solution as the diffusion coefficients (D = 1.8–2.3 × 10^−10^ m^2^ s^−1^) are all slightly smaller than that of the free ligand (D = 2.5 × 10^−10^ m^2^ s^−1^). HR-ESI-MS (acetonitrile/DMF) of the different silver(I) complexes were each dominated by a peak that was consistent with the presence of [Ag(**L**)]^+^ (*m*/*z* = 502.0661), confirming the formation of monomeric complexes in solution. Interestingly, the spectra of all the complexes also displayed minor peaks at *m*/*z* = 899.2158 indicative of the presence of [Ag(**L**)_2_]^+^ ions.

**Figure 4:**
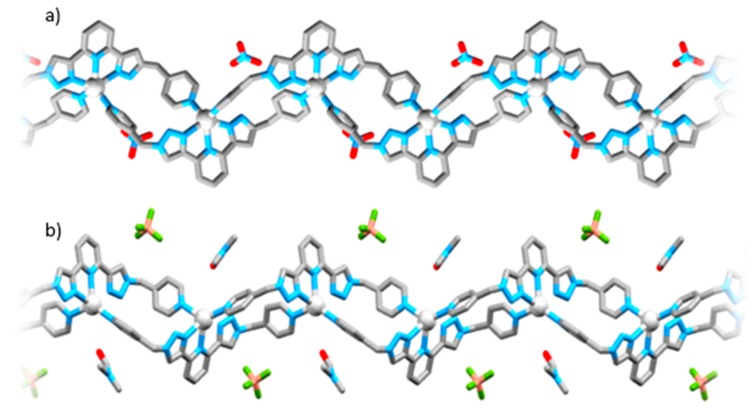
Solid state structures of (**a**) [Ag(**L**)]NO_3_ and (**b**) [Ag(**L**)]BF_4_. Selected bond lengths (Å): [Ag(**L**)]NO_3_ Ag1-N1 = 2.298, Ag1-N9 = 2.315, Ag1-N4 = 2.493, Ag1-N5 = 2.397, Ag1-N6 = 2.480; [Ag(**L**)]BF_4_ Ag1-N5 = 2.451, Ag1-N9 = 2.331, Ag1-N1 = 2.250, Ag1-N6 = 2.725, Ag1-N4 = 2.500. Color scheme: carbon, grey; nitrogen, blue; silver, silver; boron, salmon; fluorine, green; oxygen, red. Hydrogen atoms were omitted for clarity.

**Figure 5:**
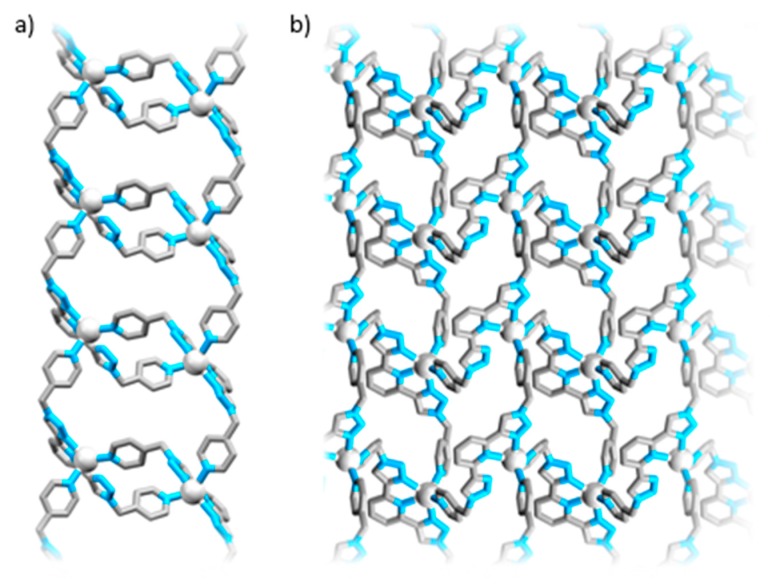
Solid state structure of [Ag(**L**)]SbF_6_ showing linear chains of macrocycles (**a**) and the extended structure (**b**). Selected bond lengths (Å): Ag1-N9 = 2.26, Ag1-N13 = 2.43, Ag1-N10 = 2.33, Ag1-N14 = 2.45, Ag1-N15 = 2.72. Color scheme: carbon, grey; nitrogen, blue; silver, silver. Hydrogen atoms, solvent molecules and SbF_6_^−^ counter anions were omitted for clarity.

All three of the silver(I) complexes were characterized by X-ray crystallography (Figure 4 and Figure 5 and Supplementary Materials, Figures S13–S15). X-ray diffraction quality crystals of [Ag(**L**)]SbF_6_ and [Ag(**L**)]BF_4_ were grown by vapour diffusion of diethyl ether into DMF solution of the complexes. Vapour diffusion of diethyl ether into DMSO and acetonitrile (2:1) solution of [Ag(**L**)] NO_3_ provided X-ray quality single crystals. Each of the silver complexes formed coordination polymers with the [Ag(**L**)]NO_3_ and [Ag(**L**)]BF_4_ compounds, crystallizing in the monoclinic space groups P2_1_/n and P2_1_/c while the [Ag(**L**)]SbF_6_ complex crystallized in the triclinic space group P$\bar{1}$. In each complex, the silver(I) ion was coordinated within the tripy tridentate pocket with two pyridyl units completing the coordination sphere giving an unsual five coordinate square pyramidal geometry (τ_5_ [36] values range from 0.085–0.20). For [Ag(**L**)]NO_3_ and [Ag(**L**)]BF_4_, this generates isostructural extended one dimensional coordination polymers with the Ag-N bond lengths (2.250–2.725 Å) in the expected ranges for Ag(I)-pyridyl [35] and Ag(I)–triazole [32,33,37] interactions (Figure 4). One Ag-N_tri_ for [Ag(**L**)]BF_4_ is quite long (Ag1-N6 = 2.725(2) Å), but the nitrogen lone pair is directly pointing to the silver(I) ion suggestive of a bonding interaction. In the linear polymers, the ligand adopts an *anti*-conformation with a pyridyl unit above and below the plane of the tripy core. In the [Ag(**L**)]BF_4_ complex the BF_4_^−^ anions hydrogen bond to the triazole C-H in a bifurcated fashion (C15---F4 3.401(4), H15---F4 2.516, C15---F1 3.304(4), H15---F1 2.502 Å), and there is no evidence of any stabilizing Ag(I)-Ag(I) interactions (the shortest Ag(I)-Ag(I) distance is 9.7707(7) Å). For the [Ag(**L**)]NO_3_ complex the NO_3_^−^ anions engage in hydrogen bonding interactions with the triazole C-H in a bifurcated fashion (C15---O1 3.266(5), H15---O1 2.306, C15---O3 3.249(5), H15---O3 2.564 Å) and with α-pyridyl hydrogens (C17---O2 3.438(5), H17---O2 2.538, C21---O1 3.165(5), H21---O1 2.456 Å of the ligand and the shortest Ag(I)-Ag(I) distance was 9.1515(6) Å.

The [Ag(**L**)]SbF_6_ complex crystallizes as a 4+4 net rather than an linear polymer (Figure 5). The ligand adopts a *syn* conformation with both pyridyl units on the same side of the plane of the tripy core. The Ag-N bond lengths (2.26–2.72 Å) are similar to those observed in the linear polymers and the network structure displays large voids but these are filled with SbF_6_^−^ anions and DMF molecules. The SbF_6_^−^ anions form hydrogen bonding interactions with the triazole C-H hydrogens (C7---F6 2.97(2), H7---F6 2.00, C17---F2 3.74(5), H17---F2 3.24 Å) and with methylene hydrogens (C16---F5 3.31(2), H16A---F5 2.94, H16B---F5 3.02) while the DMF molecules hydrogen bond to a triazole C-H (C15---O3 3.09(2), H15---O2 2.32). The shortest Ag(I)-Ag(I) distance was 10.099(2) Å (Supplementary Materials).

The [Fe(**L**)_2_](BF_4_)_2_ complex was synthesized by combining two equivalents of **L** with one equivalent of [Fe(OH_2_)_6_](BF_4_)_2_ in acetonitrile at room temperature. The solution instantly became orange (λ_max_ = 440 nm), which is consistent with the formation of the [Fe(**L**)_2_]^2+^ complex (Supplementary Materials, Figure S10). [38] ^1^H NMR spectroscopy (500 MHz, 298 K) of the [Fe(**L**)_2_](BF_4_)_2_ complex in *d*_3_-acetonitrile displayed downfield shifts of protons H_a_, H_b_, and H_c_, relative to “free” ligand while the proton resonances for H_d_, H_e_, and H_f_ are shifted upfield consistent with the formation of the expected [Fe(**L**)_2_]^2+^ complex and indicating that the iron(II) is sitting within the central tridentate tripy pocket (Supplementary Materials, Figure S11). The DOSY spectra (500 MHz, *d*_3_-acetonitrile, 298 K) of the ligand (D = 14.2 × 10^−10^ m^2^ s^−1^) and complex (D = 8.81 × 10^−10^ m^2^ s^−1^) were quite different with the diffusion coefficient of the complex just over half that of the free ligand consistent with the formation of the 2:1 octahedral complex. HR-ESI-MS (CH_3_CN) of the [Fe(**L**)_2_](BF_4_)_2_ complex only displayed one peak at *m*/*z* = 432.1435 with the correct isotope pattern and charge (2+), confirming the presence of the [Fe(**L**)_2_]^2+^ monomeric complex in solution (Supplementary Materials, Figure S12).

Interestingly, the addition of DMSO to the [Fe(**L**)_2_](BF_4_)_2_ complex results instantaneously in the loss of the orange color, providing a colorless solution as was observed previously with related [Fe(R-pytri)_2_](BF_4_)_2_ complexes. [39] ^1^H and DOSY NMR spectra (500 MHz, *d*_6_-DMSO, 298 K) are broad but the chemical shifts are the same as free **L** in DMSO indicating that the complex has dissociated into free **L** and presumably [Fe(DMSO)_6_]^2+^. [40] 

**Figure 6:**
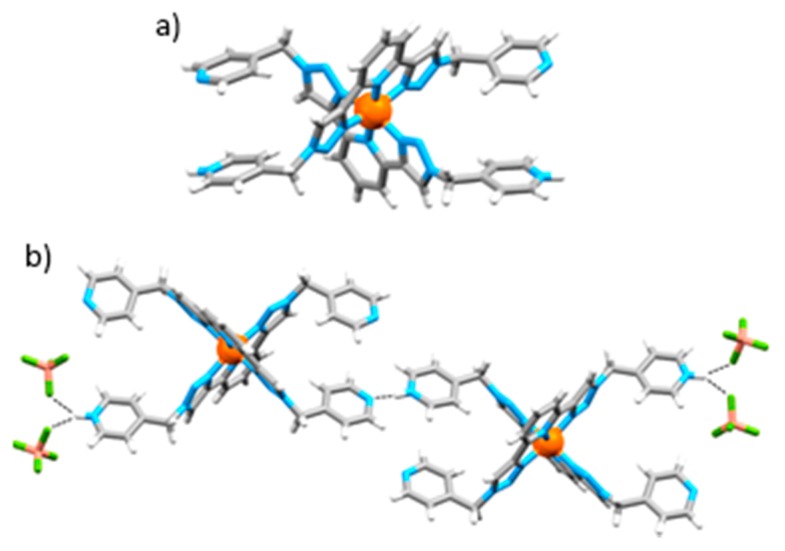
Solid state structure of [Fe(**L**)_2_](BF_4_)_2_, (**a**) [Fe(**L**)_2_(H)]^3+^ cation (**b**) hydrogen bonded dimer of [Fe(**L**)_2_(H)]. Selected bond lengths (Å): Fe1-N5 = 1.924, Fe1-N6 = 1.969, Fe1-N13 = 1.952, Fe1-N14 = 1.925, Fe1-N4 = 1.954, Fe1-N15 = 1.948, N18-H18A = 1.751, H1A-F6 = 2.254, H1A-F3 = 2.041. Color scheme: carbon, grey; nitrogen, blue; iron, orange; boron, salmon; fluorine, green; hydrogen, white. Sovlent molecules and a BF_4_^−^ anion were omitted for clarity.

X-ray diffraction quality crystals of the [Fe(**L**)_2_](BF_4_)_2_ complex were obtained by the slow diffusion of carefully layered nitromethane solutions of the ligand (**L**) and [Fe(OH_2_)_6_](BF_4_)_2_. The iron(II) complex crystallized in the triclinic space group *P*$\bar{1}$, and displayed the expected [Fe(**L**)_2_]^2+^ cation with an octahedral iron(II) center coordinated to the tripy tridentate pockets of two **L** ligands (Figure 6, Supplementary Materials, Figure S16). The Fe-N bond lengths (1.924–1.969 Å) were similar to those observed for related iron(II)-tripy [38] and pytri [39] complexes and consistent with the expected diamagnetic low spin state. The pyridyl units of the ligand adopt an *anti* conformation and interestingly two of the pyridyl units are protonated (Figure 6). One of the protonated pyridyl units engages in hydrogen bonding interactions with two of the BF_4_^−^ anions (N1---F3 2.81(1), H1A---F3 2.041, N1---F6 2.894(9), H1A---F6 2.254 Å). Two of the [Fe(**L**)_2_]^2+^ cations dimerize around another proton (N18---N18′ 2.629(5), H18A---N18 1.751) (Figure 6b).

### 2.3. Heterometallic Silver(I)-Iron(II) Complex

Having shown that silver(I) and iron(II) ions would form homometallic complexes with **L**, we attempted to generate a self-assembled heterometallic silver(I)-iron(II) coordination polymer. The [Fe(**L**)_2_](BF_4_)_2_ complex (1 equiv.) was dissolved in acetonitrile and AgBF_4_ (2 equiv.) was added at RT resulting in the formation of an orange precipitate. The orange solid was quite insoluble but could be dissolved in DMSO. However, the color immediately changed from orange to colorless consistent with decomposition of the iron(II) complex, as seen above for the [Fe(**L**)_2_](BF_4_)_2_ complex, consistently the NMR and HR-ESI-MS data were reminiscent of the [Ag(**L**)]BF_4_ complex. Elemental analysis of the orange solid was consistent with the formulation [FeAg_2_(L)_2_](BF_4_)_4_.4H_2_O.

**Figure 7:**
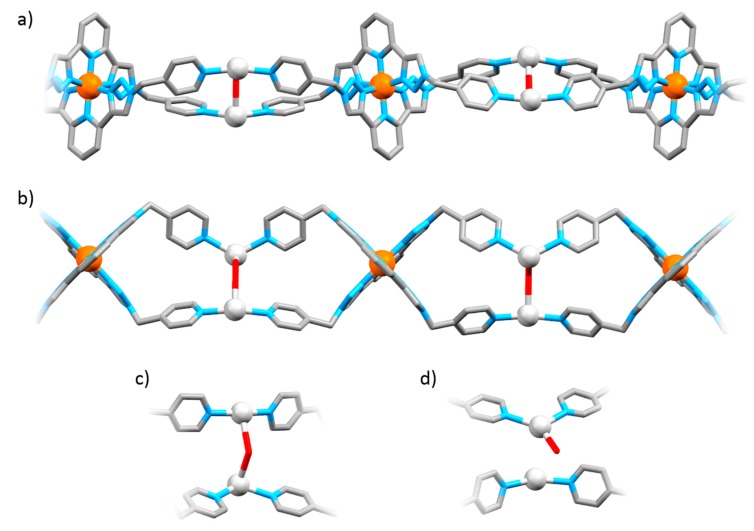
Solid state structure of the heterometallic complex [FeAg_2_(**L**)_2_](BF_4_)_4_·4H_2_O, (**a**) side view (**b**) top view (**c**,**d**) the disordered [Ag_2_(py)_4_(OH_2_)]^+^ fragments of the coordination polymer. Selected bond lengths (Å): Fe1-N4 = 1.94, Fe1-N5 = 1.93, Fe1-N6 = 1.95, Ag1A-N1A = 2.38, Ag2A-N9A = 2.22, Ag1A-O1 = 2.66, Ag2A-O1 = 2.73. Color scheme: carbon, grey; nitrogen, blue; iron, orange; oxygen, red. Solvent molecules, anions, and hydrogen atoms were omitted for clarity.

X-ray diffraction quality crystals of the heterometallic [FeAg_2_(L)_2_](BF_4_)_4_·4H_2_O complex were obtained via slow diffusion of carefully layered solutions of [Fe(**L**)_2_](BF_4_)_2_ complex in acetonitrile and AgBF_4_ in tetrahydrofuran (THF). Small orange crystals in the orthorhombic Cmca space group were obtained. While the structure displayed some disorder in the silver(I) and pyridyl fragments, it clearly showed that the heterometallic architecture had assembed (Figure 7, Supplementary Materials, Figure S17). The structure contained the expected [Fe(**L**)_2_]^2+^ cations coordinated to Ag(I) ions through the peripheral pyridyl units. The Fe-N bond lengths (1.93–1.95 Å) of the [Fe(**L**)_2_]^2+^ cations were similar to those observed in the monomeric [Fe(**L**)_2_](BF_4_)_2_ complex.

In one of the disordered silver-pyridyl components of the structure, the silver(I) ions are each coordinated to two pyridyl units (Ag-N range from 2.22 to 2.38 Å) with a water (H_2_O) ligand sandwiched between adjacent [Ag(py)_2_]^+^ units (Figure 7c). One of the silver(I) ions was essentially two coordinate with a distorted linear coordination geometry (N9A-Ag1A-N9A 164°). The other silver(I) ion was three coordinate bound to two pyridyl units and a water (H_2_O) ligand. The bond angle of the [Ag(py)_2_]^+^ units was not linear (N1A-Ag1A-N1A 121°) suggesting that the silver(I) is coordinated to a third ligand giving a trigonal coordination geometry. The silver(I) ions were both close to a H_2_O molecule (Ag1A-O1 2.66 Å, N1A-Ag1A-O1 93°, Ag2A-O1 2.67 Å) suggestive of a bridging interaction (Figure 7c). In the second disordered silver-pyridyl component of the structure the silver(I) ions are each coordinated to two pyridyl units with a water (H_2_O) ligand coordinated to only one of the [Ag(py)_2_]^+^ units (Figure 7d). There is one short silver-water interaction/bond (Ag1B-O32 2.27 Å) while the other silver ion is 3.65 Å away from the water ligand, too long for any type of bonding.

## 3. Experimental

### General

Unless otherwise stated, all of the reagents were purchased from commercial sources and used without further purification, except for 2,6-diethynylpyridine [30], which was synthesized according to the literature procedure. Solvents were laboratory reagent grade. Petroleum ether refers to the fraction of petrol boiling in the range 40–60 °C, isopropyl alcohol (IPA), methanol (CH_3_OH), dichloromethane (CH_2_Cl_2_), ethylenediaminetetraacetate (EDTA), ethynyltrimethylsilane (TMS-acetylene), acetonitrile (CH_3_CN), tetrahydrofuran (THF), dimethyl sulfoxide (DMSO), and dimethylformamide (DMF). ^1^H and ^13^C NMR spectra were recorded on either a 400 MHz Varian 400-MR or Varian 500 MHz AR spectrometer (Varian, Santa Clara, CA, USA). Chemical shifts are reported in parts per million and referenced to residual solvent peaks (CDCl_3_: ^1^H δ 7.26 ppm, ^13^C δ 77.16 ppm; CD_3_CN: ^1^H δ 1.94, ^13^C δ 1.32, 118.26 ppm, *d*_6_-DMSO: ^1^H δ 2.50 ppm; ^13^C δ 39.52 ppm, *d*_3_-nitromethane: ^1^H δ 4.30, ^13^C δ 57.3). Coupling constants (*J*) are reported in Hertz (Hz). Standard abbreviations indicating multiplicity were used as follows: m = multiplet, q = quartet, quin = quintet, t = triplet, dt = double triplet, d = doublet, dd = double doublet, s = singlet, br = broad. IR spectra were recorded on a Bruker ALPHA FT-IR spectrometer with an attached ALPHA-P measurement module (Bruker Daltonik, Bremen, Germany). Microanalyses were performed at the Campbell Microanalytical Laboratory at the University of Otago. Electrospray mass spectra (ESMS) were collected on a Bruker micrOTOF-Q spectrometer (Bruker Daltonik, Bremen, Germany).

**CAUTION**: Azides are explosive and care should be taken when handling them. Reactions were carried out on small scale. No problems were encountered during the course of this work.

*2,6-Bis(1-(pyridin-4-ylmethyl)-1H-1,2,3-triazol-4-yl)pyridine* (**L**): 4-pyridinemethanol (1.69 g, 15.5 mmol) was stirred in DMF (15 ml) in a water bath for 10 min, at which point methanesulfonyl chloride (1.77 g, 1.2 mL, 15.5 mmol) and triethylamine (1.5 g, 2.2 mL, 16 mmol) were added and the resulting orange mixture stirred for one hour. NaN_3_ (0.838 g, 12.9 mmol) was added and the mixture stirred for 18 h. 2,6-diethynylpyridine [30] (0.656 g, 5.16 mmol), CuSO_4_∙5H_2_O (0.644 g, 2.58 mmol), sodium ascorbate (1.02 g, 5.16 mmol), DMF (35 mL) and water (10 mL) were added and the mixture stirred at RT for 24 h. After addition of CH_2_Cl_2_ (75 mL) and 0.1 M EDTA/NH_4_OH aqueous solution (75 mL) and stirring for 30 min, the organic phase was washed with water (1 × 200 mL) and brine (1 × 200 mL), dried over Na_2_SO_4_, filtered and the solvent removed in vacuo. Column chromatography on silica (gradient: CH_2_Cl_2_ to acetone in 20% increments) afforded the product as an off-white powder (0.85 g, 2.2 mmol, 42%). ^1^H NMR (400 MHz, *d*_6_-DMSO, 298 k) δ: 8.75 (2H, s, H_c_), 8.58 (4H, d, *J* = 6.0 Hz, H_f_), 8.01–7.99 (3H, m, H_a,b_), 7.26 (4H, d, *J* = 6.0 Hz, H_e_), 5.79 (4H, s, H_d_). ^13^C NMR (101 MHz, *d*_6_-DMSO, 298 K) δ: 150.1(C_f_), 149.7, 147.5, 144.7, 138.4 (C_b_), 124.2 (C_c_), 122.3 (C_e_), 118.6 (C_a_), 51.8 (C_d_). HR ESI-MS (CHCl_3_/CH_3_OH) *m*/*z* = 396.1651 [M + H]^+^ (calc. for C_21_H_18_N_9_, 396.1680), 418.1478 [M + Na]^+^ (calc. for C_21_H_17_N_9_Na, 418.1499). Anal. calcd. for C_21_H_17_N_9_: C, 63.79; H, 4.33; N, 31.88%. Found: C, 63.70; H, 4.49; N, 31.82%. IR: ν (cm^−1^) 3068, 2963, 1601, 1429, 1414, 1222, 1203, 1046, 822, 784.

*[Ag(L)]NO*_3_: Vapour diffusion of diethyl ether into a solution of **L** (22 mg, 0.056 mmol) and AgNO_3_ (10 mg, 0.059 mmol) in 2:1 DMSO:acetonitrile, afforded the product as small colorless crystals which were washed with diethyl ether and dried in vacuo (29 mg, 0.051 mmol, 92%). ^1^H NMR (500 MHz, *d*_6_-DMSO, 298 K) δ: 9.07 (2H, s, H_c_), 8.60 (4H, d, *J* = 5.0 Hz, H_f_), 8.15 (1H, t, *J* = 7.8 Hz, H_a_), 8.01 (2H, d, *J* = 7.8 Hz, H_b_), 7.31 (4H, d, *J* = 6.1 Hz, H_e_), 5.85 (4H, s, H_d_). ^13^C NMR (126 MHz, *d*_6_-DMSO, 298 K) δ: 150.3 (C_f_), 147.4, 145.3, 144.5, 139.8 (C_a_), 125.2 (C_c_), 122.6 (C_e_), 120.6 (C_b_), 52.2 (C_d_). HR ESI-MS (DMSO/CH_3_CN) *m*/*z* = 502.0661 [M − NO_3_]^+^ (calc. for C_21_H_17_N_9_Ag, 502.0652). Anal. calcd. for C_21_H_17_AgN_10_O_3_·2H_2_O·1DMSO: C, 40.66; H, 4.01; N, 20.61%. Found: C, 40.68; H, 3.72; N, 20.73%. IR: ν (cm^−1^) 3067, 1601, 1577, 1421, 1333, 1012, 905, 804, 789. 

*[Ag(L)]BF*_4_: Diethyl ether was added to a DMF solution of **L** (23 mg, 0.058 mmol) and AgBF_4_ (12 mg, 0.062 mmol), affording the product as an off-white precipitate which was collected via centrifuge (13,000 rpm, 10 min) and dried in vacuo (26 mg, 0.44 mmol, 76%). ^1^H NMR (500 MHz, *d*_6_-DMSO, 298 K) δ: 9.11 (2H, s, H_c_), 8.60 (4H, d, *J* = 5.0 Hz, H_f_), 8.17 (1H, t, *J* = 7.9 Hz, H_a_), 8.01 (2H, d, *J* = 7.8 Hz H_b_), 7.31 (4H, d, *J* = 5.2 Hz, H_e_), 5.87 (4H, s, H_d_). ^13^C NMR (126 MHz, *d*_6_-DMSO, 298 K) δ: 150.3 (C_f_), 147.1, 145.0, 144.6, 139.9 (C_a_), 125.4 (C_c_), 122.6 (C_e_), 120.8 (C_b_), 52.3 (C_d_). HR ESI-MS (DMF/CH_3_CN) *m*/*z* = 502.0714 [M – BF_4_]^+^ (calc. for C_21_H_17_N_9_Ag, 502.0652). Anal. calcd. for C_21_H_17_AgN_9_BF_4_∙H_2_O: C, 41.48; H, 3.15; N, 20.73%. Found: C, 41.57; H, 2.99; N, 20.82%. IR: ν (cm^−1^) 3134, 1609, 1577, 1452, 1423, 1049, 805, 788.

*[Ag(L)]SbF*_6_: Diethyl ether was added to a DMF solution of **L** (22 mg, 0.056 mmol) and AgSbF_6_ (20 mg, 0.058 mmol), affording the product as a white precipitate which was collected via centrifuge (13,000 rpm, 10 min) and dried in vacuo (21 mg, 0.028 mmol, 50%). ^1^H NMR (500 MHz, *d*_6_-DMSO, 298 K) δ: 9.00 (2H, s, H_c_), 8.60 (4H, d, *J* = 4.4 Hz, H_f_), 8.12 (1H, t, *J* = 8.3 Hz, H_a_), 8.01 (2H, d, *J* = 7.7 Hz, H_b_), 7.30 (4H, d, *J* = 4.8 Hz, H_e_), 5.84 (4H, s, H_d_). ^13^C NMR (126 MHz, *d*_6_-DMSO, 298K) δ: 150.2 (C_f_), 147.9, 145.8, 144.5, 139.4 (C_a_), 125.0 (C_c_), 122.5 (C_e_), 120.1 (C_b_), 52.1 (C_d_). HR ESI-MS (DMF/CH_3_CN) *m*/*z* = 502.0698 [M − SbF_6_]^+^ (calc. for C_21_H_17_N_9_Ag, 502.0652). Anal. calcd. for C_21_H_17_AgF_6_N_9_Sb: C, 34.13; H, 2.32; N, 17.06%. Found C, 34.03; H, 2.36; N, 17.00%. IR: ν (cm^−1^) 3141, 1605, 1580, 1452, 1422, 1052, 807, 791, 651.

*[Fe(L)*_2_*](BF*_4_*)*_2_: Vapour diffusion of diethyl ether into an acetonitrile solution of **L** (21 mg, 0.053 mmol) and Fe(BF_4_)_2_·6H_2_O (9 mg, 0.03 mmol), afforded the product as red block crystals which were washed with diethyl ether and dried in vacuo (20 mg, 0.020 mmol, 74%). ^1^H NMR (500 MHz, CD_3_CN, 298 K) δ: 8.75 (2H, br, H_c_), 8.54 (2H, br s, H_b_), 8.43 (4H, br s, H_f_), 8.35 (1H, br, H_a_), 6.86 (4H, s, H_e_), 5.45 (4H, s, H_d_). The broadness of the peaks precluded the collection of a ^13^C NMR spectrum. HR ESI-MS (CH_3_CN) *m*/*z* = 423.1435 [M − 2BF_4_]^2+^ (calc. for C_42_H_34_FeN_18_, 423.1276). Anal. calcd. for C_42_H_34_B_2_F_8_FeN_18_∙1.25CH_3_CN·2.55H_2_O: C, 47.83; H, 3.86; N, 24.13%. Found C, 47.45; H, 3.46; N, 24.52%. IR: ν (cm^−1^) 3127, 1600, 1416, 1045, 794. UV-Vis (CH_3_CN, ε [M^−1^·cm^−1^]): λ_max_ nm = 440 (2.3 × 10^3^). 

*[FeAg*_2_*(L)*_2_*](BF*_4_*)*_4_*·4H*_2_*O*: A solution of **L** (21 mg, 0.053 mmol) and Fe(BF_4_)_2_**∙**6H_2_O (9 mg, 0.03 mmol) in acetonitrile was layered on top of a solution of AgBF_4_ (11 mg, 0.057 mmol) in THF. After diffusion small red block crystals of [Fe_n_Ag_2n_(**L**)_2n_(OH_2_)_2n_](BF_4_)_4n_ had formed, which were washed with diethyl ether and dried in vacuo (26 mg, 0.021 mmol, 79%). Anal. calcd. for C_42_H_36_Ag_2_B_4_F_16_FeN_18_O∙3H_2_O: C, 34.05; H, 2.86; N, 17.02%. Found C, 34.02; H, 2.79; N, 17.12%. IR: ν (cm^−1^) 3126, 1613, 1425, 1033, 798. 

## 4. Conclusions

A new “click” ligand, 2,6-bis(1-(pyridin-4-ylmethyl)-1*H*-1,2,3-triazol-4-yl)pyridine (**L**), featuring a tridentate tripy pocket and two pyridyl units was synthesized in modest yield (42%) using the copper(I) catalyzed azide-alkyne cycloaddition (CuAAC) reaction. The coordination chemistry of the ligand with silver(I) and iron(II) ions was examined using a range of solution and solid state techniques. When treated with silver(I) ions, the ligand forms discrete [Ag(**L**)](X) (where X = BF_4_^−^, NO_3_^−^ or SbF_6_^−^) complexes in dimethyl sulfoxide (DMSO) solution, but these complexes crystallize as coordination polymers featuring an unusual (for silver(I)) five coordinate square pyramidal geometry. With Fe(II) ions, the ligand forms a bis-ligated complex with four uncoordinated pyridyl units. The addition of AgBF_4_ to the iron(II) complex generates a heterometallic silver(I)-iron(II) linear coordination polymer. The collected results indicate that **L**, and related, hybrid “click” ligands featuring chelating pockets appended with pyridyl donors, could be exploited to generate a wide range of new metallosupramolecular architectures. A range of other octahedral metal ions could potentially be coordinated within the tripy pockets of the ligand, while other cis-protected or naked metal ions could be used to interact with the peripheral pyridyl units to generate a vast family of discrete or polymeric systems. Efforts in these directions are underway.

## Figures and Tables

**Table 1:** ^1^H DOSY data (500 MHz, 298 K) for the silver(I) and iron(II) complexes.

Compound	Diffusion Coefficient (*D*) × 10^−10^ m^2^ s^−1^	
	*d*_6_-DMSO	*d*_3_-Acetonitrile
**L**	2.5	14.2
[Ag(**L**)]NO_3_	1.8	-
[Ag(**L**)]BF_4_	2.3	-
[Ag(**L**)]SbF_6_	2.2	-
[Fe(**L**)_2_](BF_4_)_2_	2.4	8.8

## Supplementary Materials

The following are available online, ^1^H-, ^13^C-NMR, UV-Vis and HR-ESI-MS spectral data and crystallographic data in CIF format.

## References

[1] Cook T.R., Stang P.J. (2015). Recent Developments in the Preparation and Chemistry of Metallacycles and Metallacages via Coordination. Chem. Rev..

[2] Cook T.R., Zheng Y.-R., Stang P.J. (2013). Metal–Organic Frameworks and Self-Assembled Supramolecular Coordination Complexes: Comparing and Contrasting the Design, Synthesis, and Functionality of Metal–Organic Materials. Chem. Rev..

[3] He Y., Zhou W., Qian G., Chen B. (2014). Methane storage in metal-organic frameworks. Chem. Soc. Rev..

[4] Sumida K., Rogow D.L., Mason J.A., McDonald T.M., Bloch E.D., Herm Z.R., Bae T.-H., Long J.R. (2012). Carbon Dioxide Capture in Metal–Organic Frameworks. Chem. Rev..

[5] Rojas S., Devic T., Horcajada P. (2017). Metal organic frameworks based on bioactive components. J. Mater. Chem. B.

[6] Cai W., Chu C.-C., Liu G., Wáng Y.-X.J. (2015). Metal–Organic Framework-Based Nanomedicine Platforms for Drug Delivery and Molecular Imaging. Small.

[7] Liu J., Chen L., Cui H., Zhang J., Zhang L., Su C.-Y. (2014). Applications of metal-organic frameworks in heterogeneous supramolecular catalysis. Chem. Soc. Rev..

[8] Gascon J., Corma A., Kapteijn F., Llabrés i Xamena F.X. (2014). Metal Organic Framework Catalysis: Quo vadis?. ACS Catal..

[9] Cook T.R., Vajpayee V., Lee M.H., Stang P.J., Chi K.-W. (2013). Biomedical and Biochemical Applications of Self-Assembled Metallacycles and Metallacages. Acc. Chem. Res..

[10] Saha M.L., Yan X., Stang P.J. (2016). Photophysical Properties of Organoplatinum(II) Compounds and Derived Self-Assembled Metallacycles and Metallacages: Fluorescence and its Applications. Acc. Chem. Res..

[11] Xu L., Wang Y.-X., Yang H.-B. (2015). Recent advances in the construction of fluorescent metallocycles and metallocages via coordination-driven self-assembly. Dalton Trans..

[12] Croue V., Goeb S., Salle M. (2015). Metal-driven self-assembly: The case of redox-active discrete architectures. Chem. Commun..

[13] Croue V., Goeb S., Szaloki G., Allain M., Salle M. (2016). Reversible Guest Uptake/Release by Redox-Controlled Assembly/Disassembly of a Coordination Cage. Angew. Chem. Int. Ed..

[14] Bivaud S., Goeb S., Croue V., Allain M., Pop F., Salle M. (2015). Tuning the size of a redox-active tetrathiafulvalene-based self-assembled ring. Beilstein J. Org. Chem..

[15] Bivaud S., Goeb S., Croue V., Dron P.I., Allain M., Salle M. (2013). Self-Assembled Containers Based on Extended Tetrathiafulvalene. J. Am. Chem. Soc..

[16] Zhang Y.-Y., Gao W.-X., Lin L., Jin G.-X. (2017). Recent advances in the construction and applications of heterometallic macrocycles and cages. Coord. Chem. Rev..

[17] Li H., Yao Z.-J., Liu D., Jin G.-X. (2015). Multi-component coordination-driven self-assembly toward heterometallic macrocycles and cages. Coord. Chem. Rev..

[18] Laramée-Milette B., Nastasi F., Puntoriero F., Campagna S., Hanan G.S. (2017). Photo-Induced Assembly of a Luminescent Tetraruthenium Square. Chem. Eur. J..

[19] Shen C., Kennedy A.D.W., Donald W.A., Torres A.M., Price W.S., Beves J.E. (2017). Self-assembled supramolecular cages containing dinuclear ruthenium(II) polypyridyl complexes. Inorg. Chim. Acta.

[20] Yang J., Bhadbhade M., Donald W.A., Iranmanesh H., Moore E.G., Yan H., Beves J.E. (2015). Self-assembled supramolecular cages containing ruthenium(II) polypyridyl complexes. Chem. Commun..

[21] Schouwey C., Holstein J.J., Scopelliti R., Zhurov K.O., Nagornov K.O., Tsybin Y.O., Smart O.S., Bricogne G., Severin K. (2014). Self-Assembly of a Giant Molecular Solomon Link from 30 Subcomponents. Angew. Chem. Int. Ed..

[22] Iranmanesh H., Arachchige K.S.A., Bhadbhade M., Donald W.A., Liew J.Y., Liu K.T.C., Luis E.T., Moore E.G., Price J.R., Yan H. (2016). Chiral Ruthenium(II) Complexes as Supramolecular Building Blocks for Heterometallic Self-Assembly. Inorg. Chem..

[23] Beves J.E., Constable E.C., Decurtins S., Dunphy E.L., Housecroft C.E., Keene T.D., Neuberger M., Schaffner S., Zampese J.A. (2009). Structural diversity in the reactions of 4′-(pyridyl)-2,2′:6′,2″-terpyridine ligands and bis{4-(4-pyridyl)-2,2′:6′,2″-terpyridine}iron(II) with copper(II) salts. CrystEngComm.

[24] Beves J.E., Dunphy E.L., Constable E.C., Housecroft C.E., Kepert C.J., Neuburger M., Price D.J., Schaffner S. (2008). Vectorial property dependence in bis{4′-(*n*-pyridyl)-2,2″:6′,2″-terpyridine}iron(ii) and ruthenium(ii) complexes with n = 2, 3 and 4. Dalton Trans..

[25] Beves J.E., Bray D.J., Clegg J.K., Constable E.C., Housecroft C.E., Jolliffe K.A., Kepert C.J., Lindoy L.F., Neuburger M., Price D.J., Schaffner S., Schaper F. (2008). Expanding the 4,4′-bipyridine ligand: Structural variation in {M(pytpy)2}^2+^ complexes (pytpy = 4′-(4-pyridyl)-2,2′:6′,2″-terpyridine, M = Fe, Ni, Ru) and assembly of the hydrogen-bonded, one-dimensional polymer. Inorg. Chim. Acta.

[26] Beves J.E., Constable E.C., Housecroft C.E., Neuburger M., Schaffner S. (2007). A palladium(II) complex of 4′-(4-pyridyl)-2,2′:6′,2″-terpyridine: Lattice control through an interplay of stacking and hydrogen bonding effects. Inorg. Chem. Commun..

[27] Ollagnier C.M.A., Nolan D., Fitchett C.M., Draper S.M. (2012). 4′-(pyridyl)-2,2′:6′,2″-Terpyridine ligands: Discrete metal complexes and their polymeric assemblies as a function of N-pyridyl substitution patterns. Supramol. Chem..

[28] Ollagnier C.M., Nolan D., Fitchett C.M., Draper S.M. (2007). [Ru(3-pyridyl-4′-terpy)2]^2+^ as a metallo-ligand—Adding to the complexity of supramolecular polymers. Inorg. Chem. Commun..

[29] Vasdev R.A.S., Preston D., Crowley J.D. (2017). Functional metallosupramolecular architectures using 1,2,3-triazole ligands: It’s as easy as 1,2,3 “click”. Dalton Trans..

[30] Dana B.H., Robinson B.H., Simpson J. (2002). Intramolecular interactions in 2,6-pyridylacetylenes and their Co2(CO)4dppm complexes. J. Organomet. Chem..

[31] Bevilacqua V., King M., Chaumontet M., Nothisen M., Gabillet S., Buisson D., Puente C., Wagner A., Taran F. (2014). Copper-Chelating Azides for Efficient Click Conjugation Reactions in Complex Media. Angew. Chem. Int. Ed..

[32] Crowley J.D., Bandeen P.H., Hanton L.R. (2010). A one pot multi-component CuAAC “click” approach to bidentate and tridentate pyridyl-1,2,3-triazole ligands: Synthesis, x-ray structures and copper(II) and silver(I) complexes. Polyhedron.

[33] Crowley J.D., Bandeen P.H. (2010). A multicomponent CuAAC “click” approach to a library of hybrid polydentate 2-pyridyl-1,2,3-triazole ligands: New building blocks for the generation of metallosupramolecular architectures. Dalton Trans..

[34] Preston D., Tucker R.A.J., Garden A.L., Crowley J.D. (2016). Heterometallic [M_n_Pt_n_(L)_2n_]^x+^ Macrocycles from Dichloromethane-Derived Bis-2-pyridyl-1,2,3-triazole Ligands. Inorg. Chem..

[35] Kilpin K.J., Gower M.L., Telfer S.G., Jameson G.B., Crowley J.D. (2011). Toward the Self-Assembly of Metal-Organic Nanotubes Using Metal-Metal and π-Stacking Interactions: Bis(pyridylethynyl) Silver(I) Metallo-macrocycles and Coordination Polymers. Inorg. Chem..

[36] Addison A.W., Rao T.N., Reedijk J., van Rijn J., Verschoor G.C. (1984). Synthesis, structure, and spectroscopic properties of copper(II) compounds containing nitrogen-sulphur donor ligands; the crystal and molecular structure of aqua[1,7-bis(*N*-methylbenzimidazol-2′-yl)-2,6-dithiaheptane]copper(II) perchlorate. J. Chem. Soc. Dalton Trans..

[37] Gower M.L., Crowley J.D. (2010). Self-assembly of silver(I) metallomacrocycles using unsupported 1,4-substituted-1,2,3-triazole “click” ligands. Dalton Trans..

[38] Li Y., Huffman J.C., Flood A.H. (2007). Can terdentate 2,6-bis(1,2,3-triazol-4-yl)pyridines form stable coordination compounds?. Chem. Commun..

[39] Vellas S.K., Lewis J.E.M., Shankar M., Sagatova A., Tyndall J.D.A., Monk B.C., Fitchett C.M., Hanton L.R., Crowley J.D. (2013). [Fe_2_L_3_]^4+^ cylinders derived from bis(bidentate) 2-pyridyl-1,2,3-triazole “click” ligands: Synthesis, structures and exploration of biological activity. Molecules.

[40] White A.P., Robertson K.N., Cameron T.S., Liengme B.V., Leznoff D.B., Trudel S., Aquino M.A.S. (2007). Synthesis and characterization of [M(DMSO)_6_][SnCl_6_] complexes (M = Fe^2+^, Co^2+^, and Ni^2+^)—An old mystery solved. Can. J. Chem..

