# Supplementary material for: Self-Assembly with 2,6-Bis(1-(pyridin-4-ylmethyl)-1H-1,2,3-triazol-4-yl)pyridine: Silver(I) and Iron(II) Complexes
Source: Molecules. 2017 Oct 19;22(10):1762. doi: 10.3390/molecules22101762 (PMC6151823; doi:10.3390/molecules22101762)
Supplement: Supplementary file 1 [file molecules-22-01762-s001.pdf]

Supporting Information

Self-assembly with 2,6-bis(1-(pyridin-4-ylmethyl)-1H-1,2,3-triazol-4-yl)pyridine: silver(I) and iron(II) complexes

Daniel A. W. Ross,[1] Dan Preston,[1,*] and James D. Crowley[1,*]

[1]Department of Chemistry, University of Otago, PO Box 56, Dunedin, New Zealand.

* jcrowley@chemistry.otago.ac.nz
daniel.preston@otago.ac.nz

Contents

1 Experimental 3

1.1 General 3

1.2 Spectral data 4

2 X-ray Data 10

2.1 $[Ag_n(L)_n](NO_3)_n$ 10

2.2 $[Ag_n(L)_n](BF_4)_n \cdot 3DMF$ 11

2.3 $[Ag_n(L)_n](SbF_6)_n \cdot 4nDMF$ 12

2.4 $H_{1.5}[Fe(L)_2](BF_4)_{3.5} \cdot 3CH_3NO_2$ 13

2.5 $[Fe_nAg_{2n}(L)_{2n}(OH_2)_{2n}](BF_4)_{4n}$ 14

2.6 Crystallographic data 15

3 References 17

1 Experimental

1.1 General

Unless otherwise stated, all reagents were purchased from commercial sources and used without further purification, except for 2, 6- diethynylpyridine[1] which was synthesised according to literature procedures. Solvents were laboratory reagent grade. Petroleum ether refers to the fraction of petrol boiling in the range 40-60 °C, isopropyl alcohol (IPA), methanol (MeOH), dichloromethane (CH_2Cl_2), ethylenediaminetetraacetate (EDTA), ethynyltrimethylsilane (TMS-acetylene), tetrahydrofuran (THF), dimethyl sulfoxide (DMSO), dimethylformamide (DMF). ^{1}H and ^{13}C NMR spectra were recorded on either a 400 MHz Varian 400-MR or Varian 500 MHz AR spectrometer. Chemical shifts are reported in parts per million and referenced to residual solvent peaks ($CDCl_3$: ^{1}H δ 7.26 ppm, ^{13}C δ 77.16 ppm; CD_3CN: ^{1}H δ 1.94, ^{13}C δ 1.32, 118.26 ppm, d_6-DMSO: ^{1}H δ 2.50 ppm; ^{13}C δ 39.52 ppm, d_3-nitromethane: ^{1}H δ 4.30, ^{13}C δ 57.3). Coupling constants (*J*) are reported in Hertz (Hz). Standard abbreviations indicating multiplicity were used as follows: m = multiplet, q = quartet, quin = quintet, t = triplet, dt = double triplet, d = doublet, dd = double doublet, s = singlet, br = broad. IR spectra were recorded on a Bruker ALPHA FT-IR spectrometer with an attached ALPHA-P measurement module. Microanalyses were performed at the Campbell Microanalytical Laboratory at the University of Otago. Electrospray mass spectra (ESMS) were collected on a Bruker micrOTOF-Q spectrometer.

CAUTION: Azides are explosive and care should be taken when handling them. Reactions were carried out on small scale. No problems were encountered during the course of this work.

1.2 Spectral data

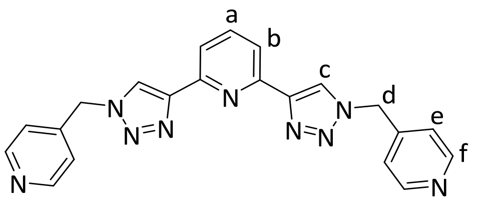

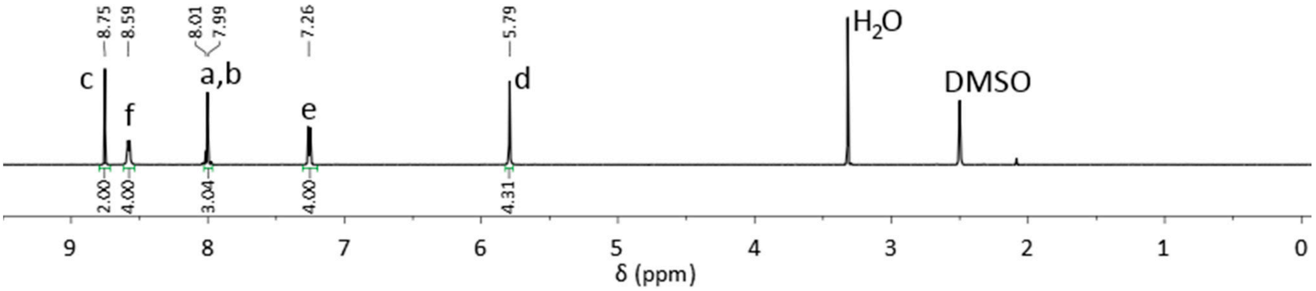

Figure S1 ^{1}H NMR (400 MHz, d_6-DMSO, 298 K) of **L**.

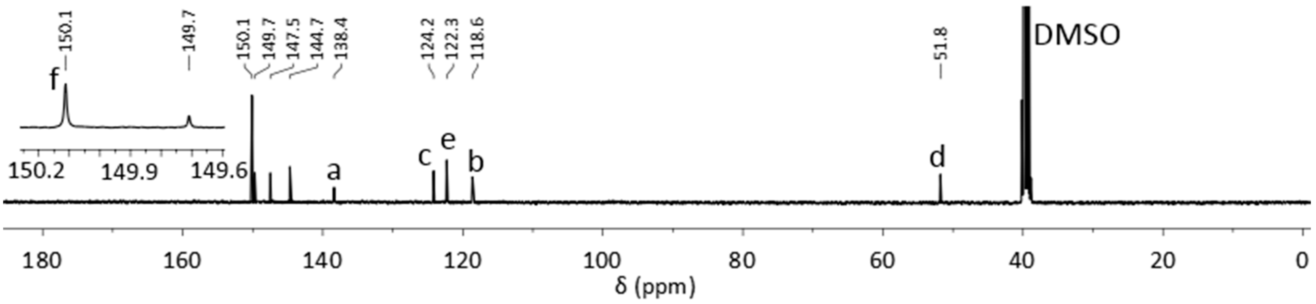

Figure S2 ^{13}C NMR (101 MHz, d_6-DMSO, 298 K) of **L**.

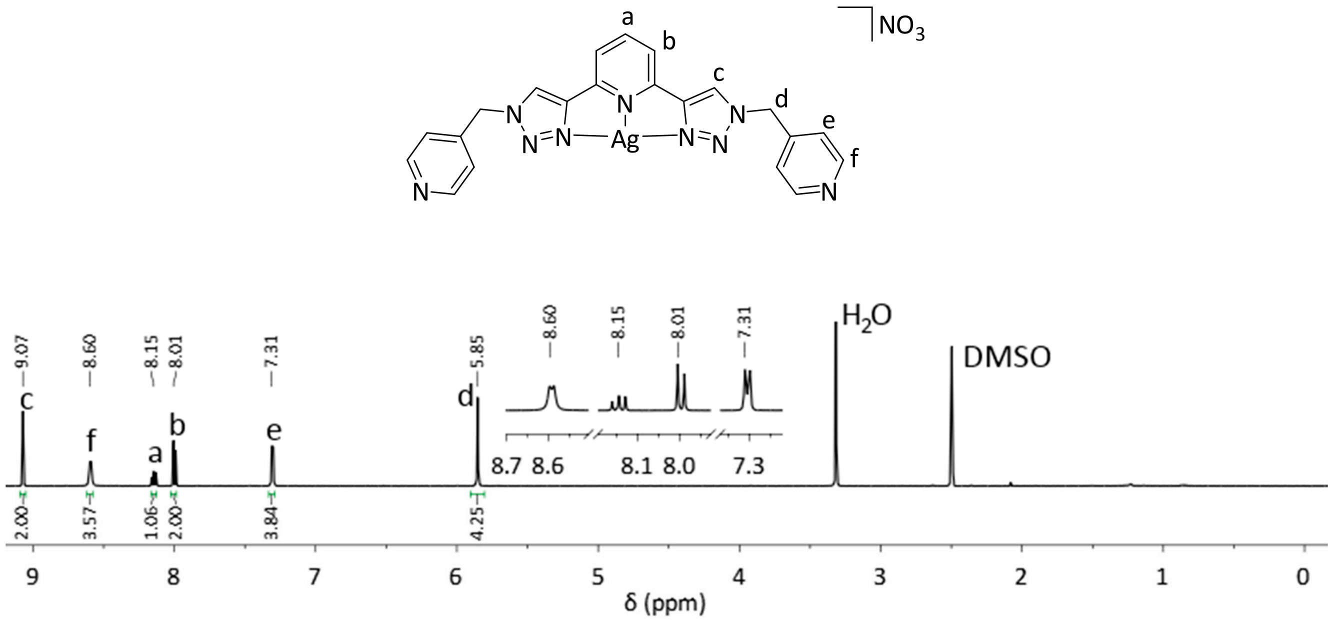

Figure S3 ^{1}H NMR (500 MHz, d_6-DMSO, 298 K) of [Ag(**L**)]NO_3.

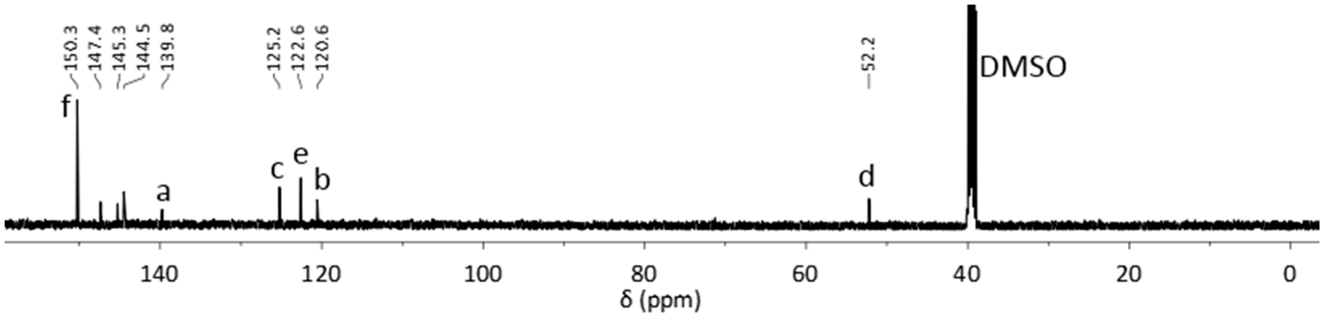

Figure S4 ^{13}C NMR (126 MHz, d_6-DMSO, 298 K) of [Ag(**L**)]NO_3.

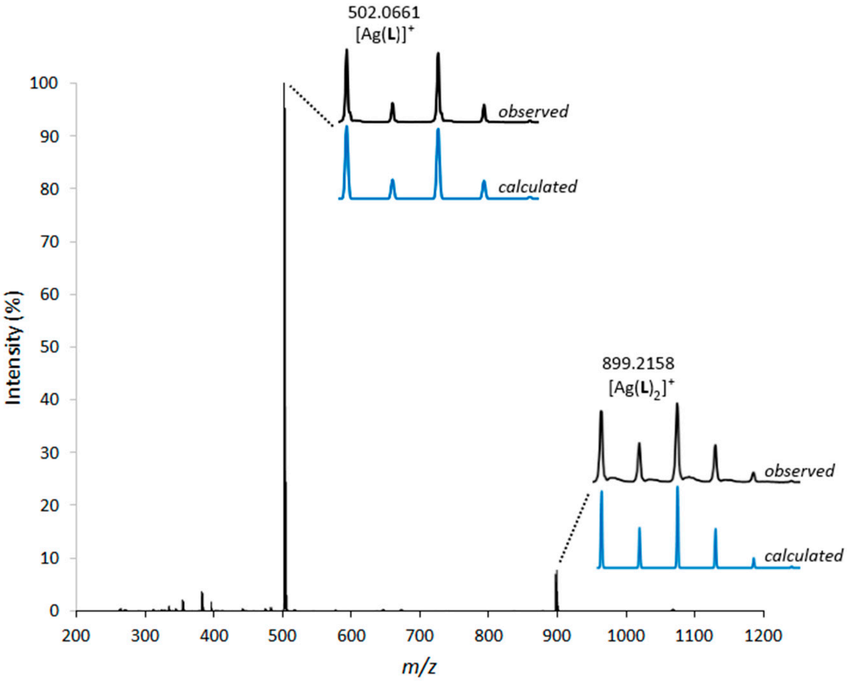

Figure S5 HR ESI-MS (DMF/CH_3CN) of [Ag(**L**)]NO_3.

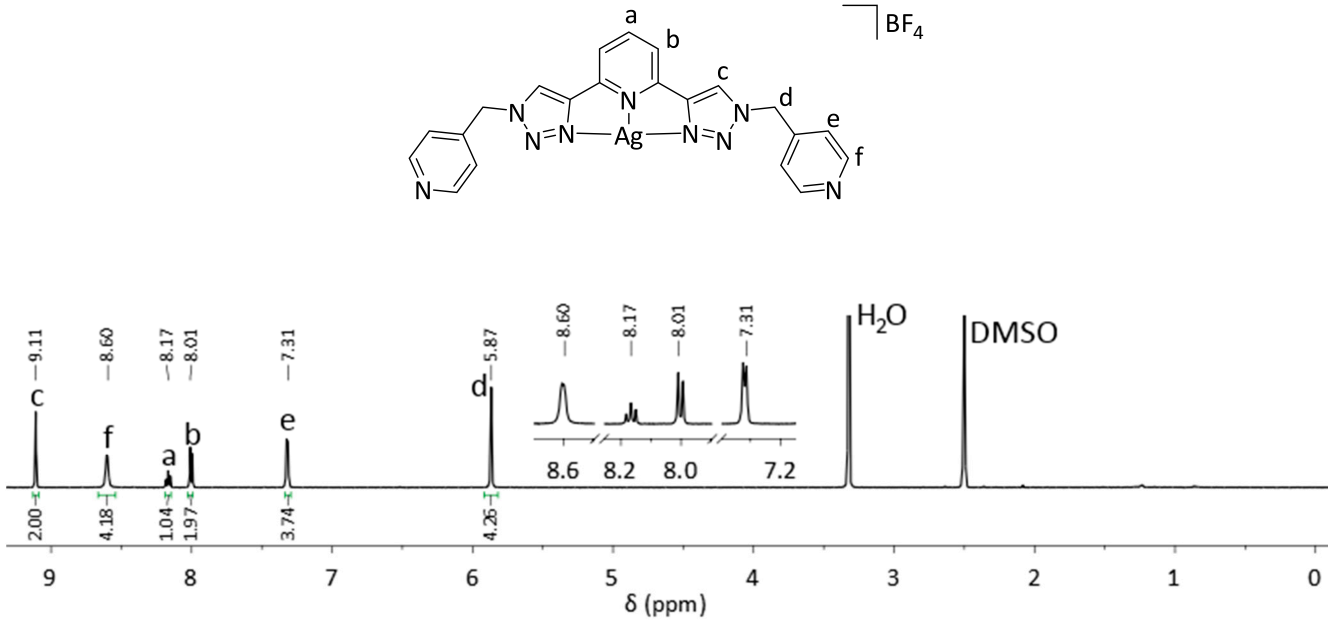

Figure S6 ^{1}H NMR (500 MHz, d_6-DMSO, 298 K) of [Ag(**L**)]BF_4.

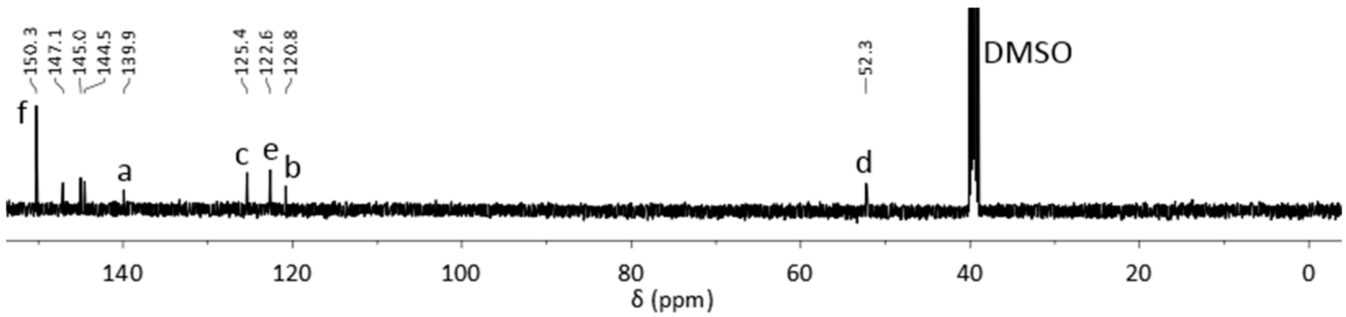

Figure S7 ^{13}C NMR (126 MHz, d_6-DMSO, 298 K) of [Ag(**L**)]BF_4.

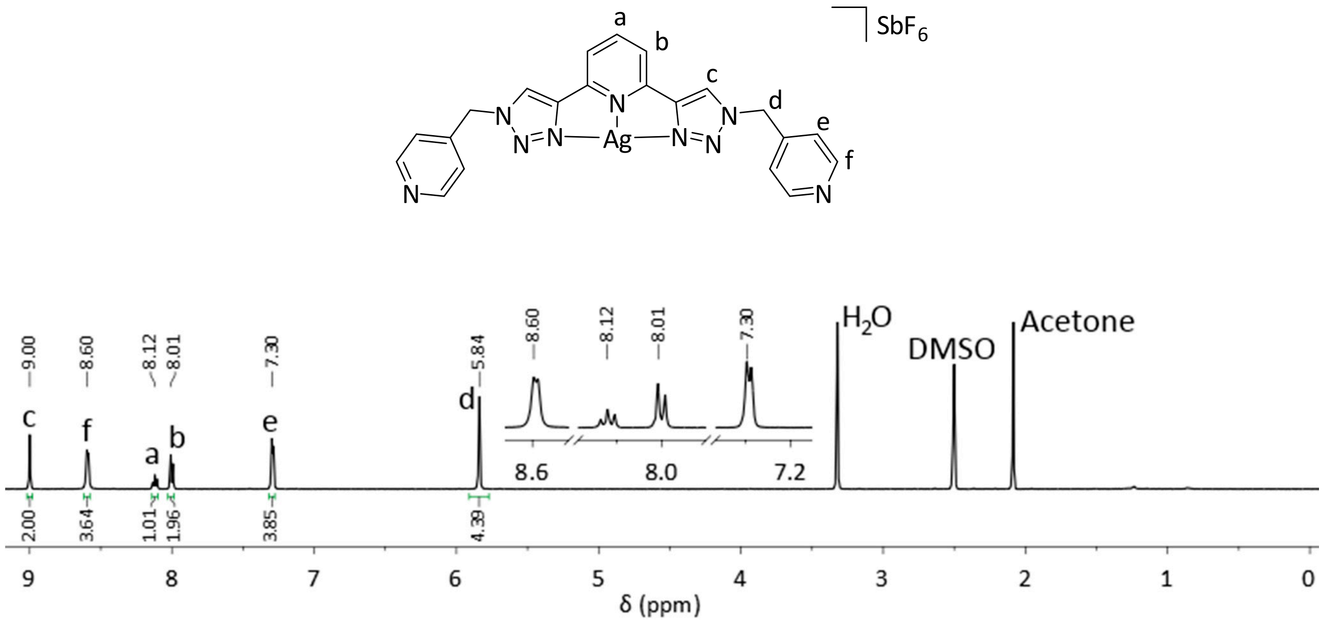

Figure S8 ^{1}H NMR (500 MHz, d_6-DMSO, 298 K) of [Ag(**L**)]SbF_6.

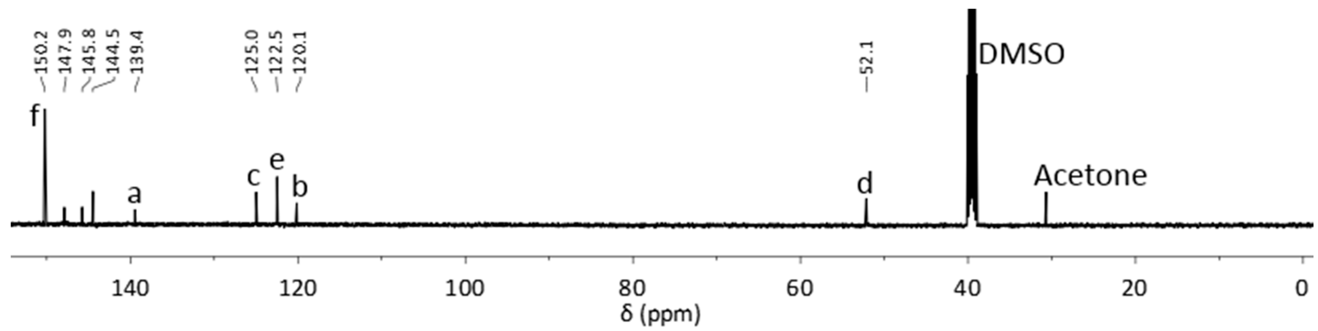

Figure S9 ^{13}C NMR (126 MHz, d_6-DMSO, 298 K) of [Ag(**L**)]SbF_6.

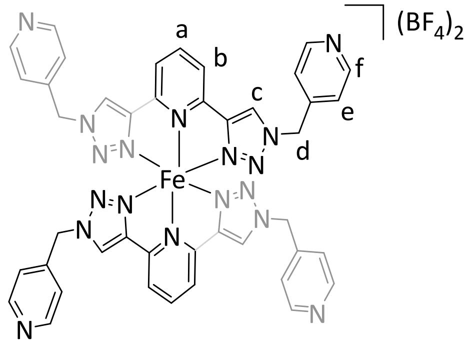

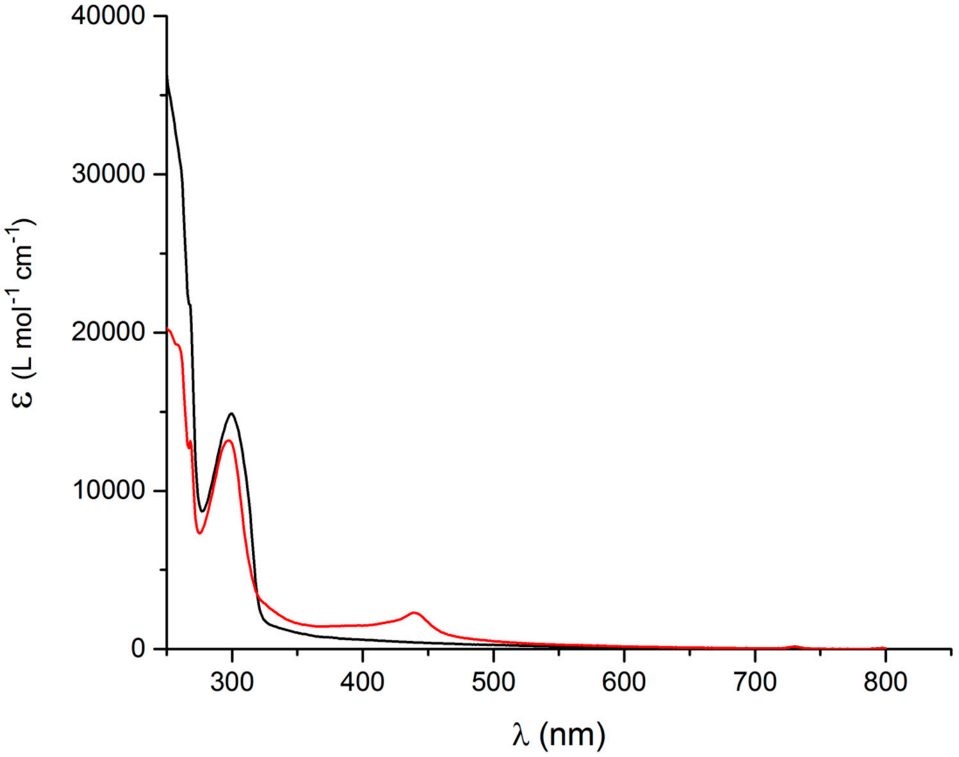

Figure S10 UV-Visible spectra (CH_3CN) of $[Fe(\mathbf{L})_2](BF_4)_2$ (red) and **L** (black).

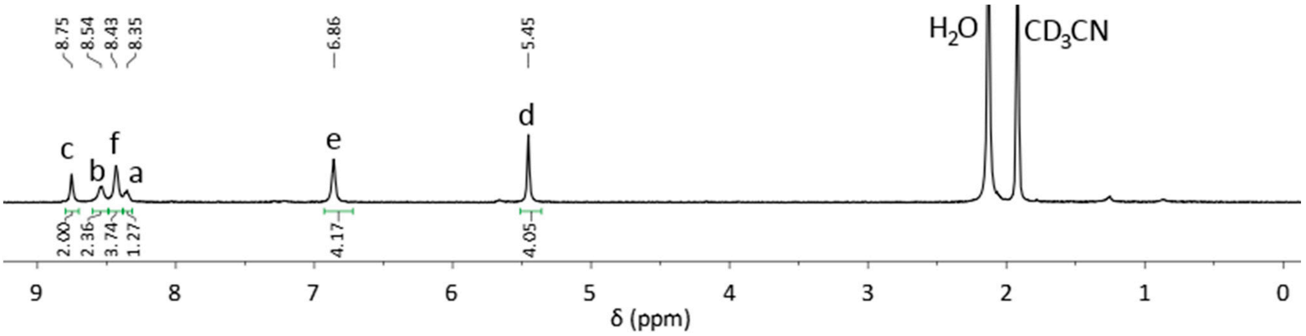

Figure S11 ^1H NMR (500 MHz, CD_3CN, 298 K) of $[Fe(\mathbf{L})_2](BF_4)_2$

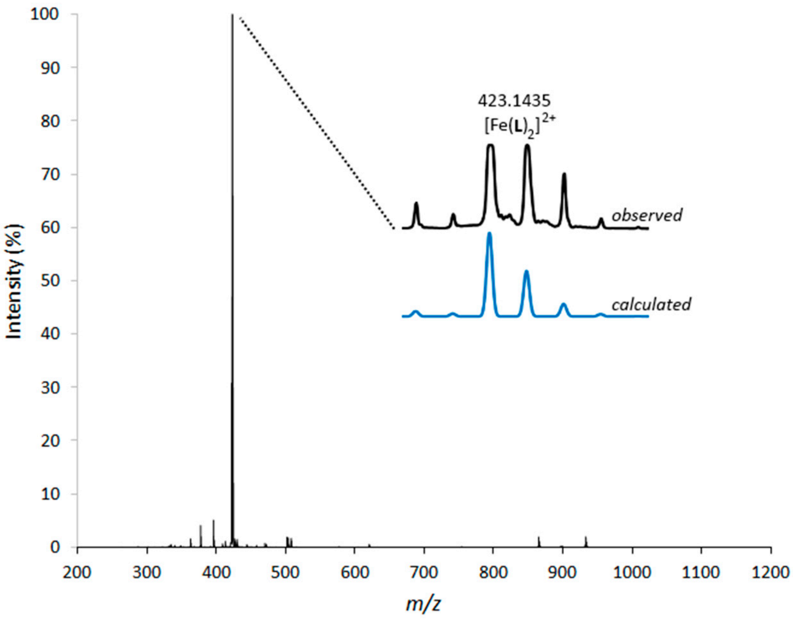

Figure S12 HR ESI-MS (DMF/CH_3CN) of [Fe(**L**)$_2$](BF_4)$_2$.

2 X-ray Data

2.1 $[Ag_n(L)_n](NO_3)_n$

CCDC #: 1576506. Vapour diffusion of diethyl ether into a 1:1 solution of **L** and $AgNO_3$ in acetonitrile/DMSO resulted in the formation of long, colourless needle crystals of $[Ag_n(\mathbf{L})_n](NO_3)_n$. X-ray data were collected at 100 K on an Agilent Technologies Supernova system using Cu Kα radiation with exposures over 1.0°, and data were treated using CrysAlisPro[2] software. The structure was solved using SHELXS within the X-Seed package[3] and weighted full-matrix refinement on F^2 was carried out using SHELXL-97[4] running within the WinGX package.[5] All non-hydrogen atoms were refined anisotropically. Hydrogen atoms attached to carbons were placed in calculated positions and refined using a riding model. The structure was solved in the body-centred space group $P2_1/c$ and refined to an R_1 value of 5.3%. The asymmetric unit contains one $[Ag(\mathbf{L})](NO_3)$ subunit.

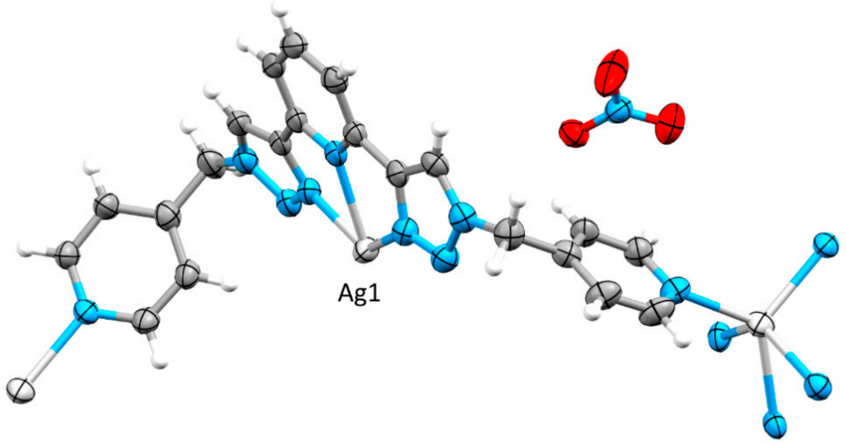

Figure S13 Mercury ellipsoid plot of the asymmetric unit of $[Ag_n(\mathbf{L})_n](NO_3)_n$. Ellipsoids are shown at the 50% probability level. Color scheme: carbon grey, hydrogen white, nitrogen blue, oxygen red, silver silver.

There was diffuse electron density within the lattice that could not be appropriately modelled. We assign this density to 4 DMSO solvent molecules (42 electrons each), 2 within each void space. The SQUEEZE routine running from within PLATON was employed to resolve the diffuse electron density.

Table S1 SQUEEZE details for $[Ag_n(\mathbf{L})_n](NO_3)_n$.

Void	x	y	z	Volume	Electrons
**1**	0.000	0.500	0.000	359	83
**2**	0.000	0.000	0.500	359	83

2.2 $[Ag_n(\mathbf{L})_n](BF_4)_n \cdot 3DMF$

CCDC #: 1576507. Vapour diffusion of diethyl ether into a 1:1 solution of **L** and $AgBF_4$ in DMF resulted in the formation of long, colourless needle crystals of $[Ag_n(\mathbf{L})_n](BF_4)_n \cdot 3nDMF$. X-ray data were collected at 100 K on an Agilent Technologies Supernova system using Cu Kα radiation with exposures over 1.0°, and data were treated using CrysAlisPro[2] software. The structure was solved using SHELXS within the X-Seed package[3] and weighted full-matrix refinement on F^2 was carried out using SHELXL-97[4] running within the WinGX package.[5] All non-hydrogen atoms were refined anisotropically. Hydrogen atoms attached to carbons were placed in calculated positions and refined using a riding model. The structure was solved in the body-centred space group $P2_1/n$ and refined to an R_1 value of 4.3%. The asymmetric unit contains one $[Ag(\mathbf{L})](BF_4) \cdot 3DMF$ subunit.

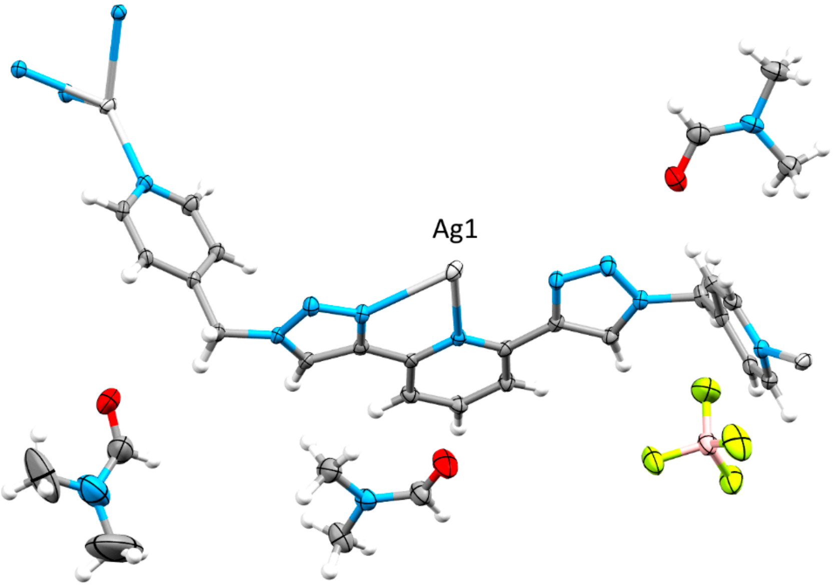

Figure S14 Mercury ellipsoid plot of the asymmetric unit of $[Ag_n(\mathbf{L})_n](BF_4)_n \cdot 3nDMF$. Ellipsoids are shown at the 50% probability level. Color scheme: carbon grey, hydrogen white, boron salmon, fluorine yellow, nitrogen blue, oxygen red, silver silver.

2.3 $[Ag_n(L)_n](SbF_6)_n\cdot 4nDMF$

CCDC #: 1576508. Vapour diffusion of diethyl ether into a 1:1 solution of **L** and $AgSbF_6$ in DMF resulted in the formation of long, colourless needle crystals of $[Ag_n(\mathbf{L})_n](SbF_6)_n\cdot 4nDMF$. X-ray data were collected at 100 K on an Agilent Technologies Supernova system using Cu Kα radiation with exposures over 1.0°, and data were treated using CrysAlisPro[2] software. The structure was solved using SHELXS within the X-Seed package[3] and weighted full-matrix refinement on F^2 was carried out using SHELXL-97[4] running within the WinGX package.[5] All non-hydrogen atoms were refined anisotropically. Hydrogen atoms attached to carbons were placed in calculated positions and refined using a riding model. The structure was solved in the body-centred space group $P\overline{1}$ and refined to an R_1 value of 10.3%. The asymmetric unit contains one $[Ag(\mathbf{L})](BF_4)\cdot 4DMF$ subunit.

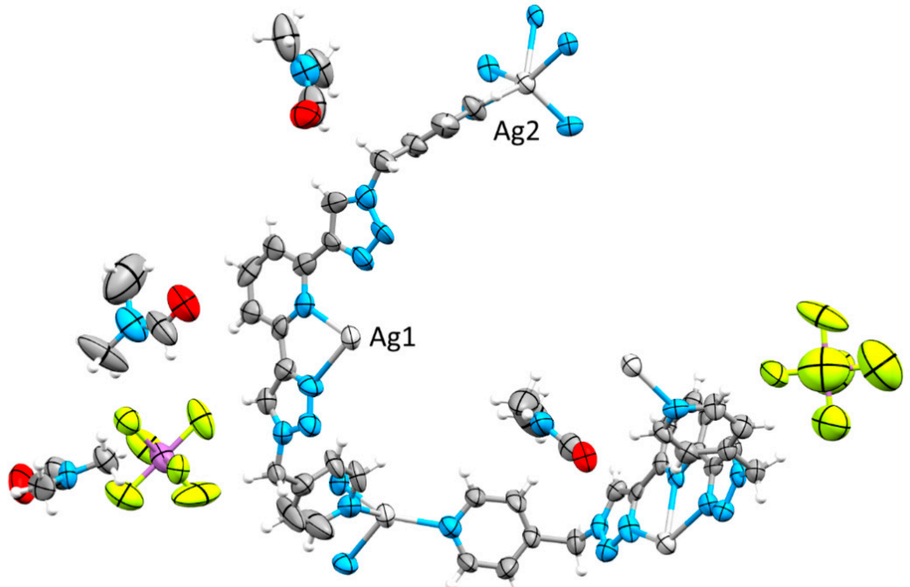

Figure S15 Mercury ellipsoid plot of the asymmetric unit of $[Ag_n(\mathbf{L})_n](SbF_6)_n\cdot 4nDMF$. Ellipsoids are shown at the 50% probability level. Color scheme: carbon grey, hydrogen white, antimony violet, fluorine yellow, nitrogen blue, oxygen red, silver silver.

Disorder in the structure was modelled using the SADI, ISOR, SIMU and DFIX commands.

2.4 $H_{1.5}[Fe(L)_2](BF_4)_{3.5}\cdot 3CH_3NO_2$

CCDC #: 1576509. Careful layering of $Fe(BF_4)_2\cdot 6H_2O$ and **L** in nitromethane and diffusion resulted in the formation of red block crystals of $H_{1.5}[Fe(\mathbf{L})_2](BF_4)_{3.5}\cdot 3CH_3NO_2$. X-ray data were collected at 100 K on an Agilent Technologies Supernova system using Cu Kα radiation with exposures over 1.0°, and data were treated using CrysAlisPro[2] software. The structure was solved using SHELXT and weighted full-matrix refinement on F^2 was carried out using SHELXL-97[4] running within the WinGX package.[5] All non-hydrogen atoms were refined anisotropically. Hydrogen atoms attached to carbons were placed in calculated positions and refined using a riding model. The structure was solved in the hexagonal space group $P\bar{1}$ and refined to an R_1 value of 10.9%. The asymmetric unit contains one Fe(II) metal ion, two ligands **L**, 3.5 BF_4^- anions, 1.5 protons (on pendant pyridine rings) and 3 nitromethane solvent molecules.

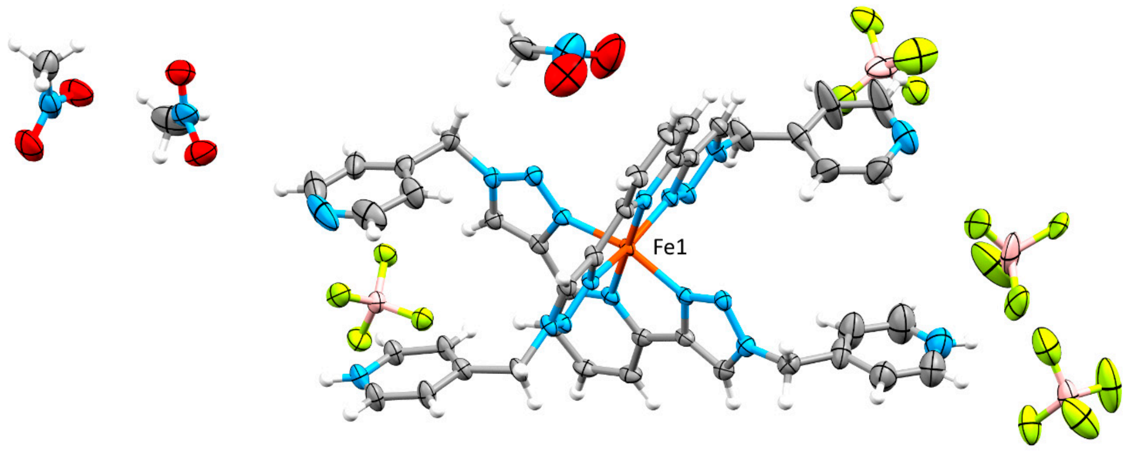

Figure S16 Mercury ellipsoid plot of the asymmetric unit of $H_{1.5}[Fe(\mathbf{L})_2](BF_4)_{3.5}\cdot 3CH_3NO_2$. Ellipsoids shown at 50% probability level. Color scheme: carbon grey, hydrogen white, boron salmon, fluorine yellow, iron orange, nitrogen blue, oxygen red.

Disorder in the structure was modelled using the SADI, SIMU and ISOR commands. There was diffuse electron density (98 electrons) in the lattice that could not be appropriately modelled. The SQUEEZE routine running within PLATON was employed to resolve this problem. We assign this electron density to 2 solvent water molecules within 2 voids (2 x 10 electrons) and 2 lots of 1 nitromethane and 1 water molecules (32 and 10 electrons for 42 electrons total) each within one of another two voids, for 104 total electrons.

Table S2 SQUEEZE details for $H_{1.5}[Fe(\mathbf{L})_2](BF_4)_{3.5}\cdot 3CH_3NO_2$**9**.

Void	x	y	z	Volume	Electrons
**1**	0.002	0.337	0.931	15	8
**2**	-0.003	0.663	0.069	14	8
**3**	0.219	0.095	0.366	70	40
**4**	0.309	0.211	0.067	18	1
**5**	0.691	0.789	0.933	18	1
**6**	0.781	0.905	0.634	70	40

2.5 $[Fe_nAg_{2n}(\mathbf{L})_{2n}(OH_2)_{2n}](BF_4)_{4n}$

CCDC #: 1576510. Layering of a solution of $[Fe(\mathbf{L})_2](BF_4)_2$ in acetonitrile above a solution of $AgBF_4$ in THF and diffusion resulted in the formation of red block crystals of $[Fe_nAg_{2n}(\mathbf{L})_{2n}(OH_2)_{2n}](BF_4)_{4n}$. X-ray data were collected at 100 K on an Agilent Technologies Supernova system using Cu Kα radiation with exposures over 1.0°, and data were treated using CrysAlisPro[2] software. The structure was solved using SHELXT and weighted full-matrix refinement on F^2 was carried out using SHELXL-97[4] running within the WinGX package.[5] All non-hydrogen atoms were refined anisotropically. Hydrogen atoms attached to carbons were placed in calculated positions and refined using a riding model. The structure was solved in the monoclinic space group *Cmca* and refined to an R_1 value of 9.2%. The asymmetric unit contains 0.5 Fe(II) and 1 Ag(I) cations, 1 ligand **L**, 1 oxygen atom and 1.5 BF_4^- counterions.

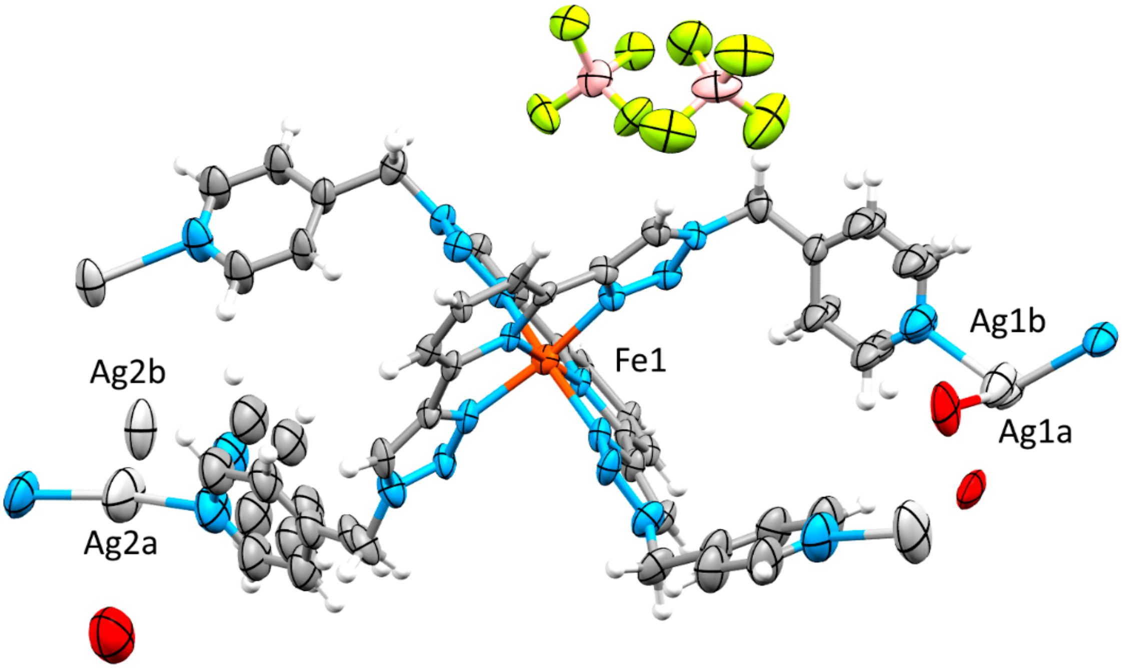

Figure S17 Mercury ellipsoid plot of the asymmetric unit of $[Fe_nAg_{2n}(\mathbf{L})_{2n}OH_2)_{2n}](BF_4)_{4n}$. Ellipsoids shown at 50% probability level. Color scheme: carbon grey, hydrogen white, boron salmon, fluorine yellow, iron orange, nitrogen blue, oxygen red, silver silver. Hydrogen atoms not shown on oxygen atoms.

The crystals were highly prone to desolvation, even on the loop under the nitrogen stream. As such there was a necessary compromise between targeted resolution and number of frames. This particular data set was the best of multiple attempts. Disorder within the structure was modelled using the SADI, FLAT and DFIX commands. The two silver(I) cations, coordinated oxygen atoms and coordinated pendant pyridyl rings were disordered over two sites and were modelled using the PART command. There was diffuse electron density within the lattice (4 x 251 electrons = 1004 electrons) that could not be appropriately modelled and the SQUEEZE command running within PLATON was employed to resolve this issue. We assign this electron density to the four missing BF_4^- counterions and assorted solvent molecules.

Table S3 SQUEEZE details for $[Fe_nAg_{2n}(\mathbf{L})_{2n}(OH_2)_{2n}](BF_4)_{4n}$.

Void	x	y	z	Volume	Electrons
**1**	0.000	0.013	0.000	833	251
**2**	0.500	-0.002	0.000	833	251
**3**	0.000	0.400	0.500	833	251
**4**	0.500	0.900	0.500	833	251

2.6 Crystallographic data

Identification code	$[Ag_n(L)_n](NO_3)_n$		$[Ag_n(L)_n](BF_4)_n\cdot 3nDMF$		$[Ag_n(L)_n](SbF_6)_n\cdot 4nDMF$	
**Empirical formula**	$C_{21}H_{17}AgN_{10}O_3$		$C_{30}H_{38}AgBF_4N_{12}O_3$		$C_{54}H_{62}Ag_2F_{12}N_{22}O_4Sb_2$	
**Formula weight**	565.32		809.4		1770.49	
**Temperature**	99.97(10) K		100(2) K		100.00(10) K	
**Wavelength**	1.54184 Å		0.71073 Å		1.54184 Å	
**Crystal system**	Monoclinic		Monoclinic		Triclinic	
**Space group**	$P2_1/_c$		$P2_1/n$		$P\bar{1}$	
**Unit cell dimensions**	a = 8.4354(4) Å	α= 90°	a = 7.6033(3) Å	α= 90°	a = 10.0993(4) Å	α= 91.355(4)°
	b = 18.8604(9) Å	β= 91.064(5)°	b = 25.8625(10) Å	β= 93.513(4)°	b = 12.9821(6) Å	β= 93.148(3)°
	c = 17.5483(7) Å	γ = 90°	c = 18.3349(8) Å	γ = 90°	c = 25.1748(10) Å	γ = 90.745(4)°
**Volume**	2791.4(2) $Å^3$		3598.6(3) $Å^3$		3294.4(2) $Å^3$	
**Z**	4		4		2	
**Density (calculated)**	1.345 mg/m^3		1.494 mg/m^3		1.785 mg/m^3	
**Absorption coefficient**	6.125 mm^{-1}		0.631 mm^{-1}		11.977 mm^{-1}	
**F(000)**	1136		1656		1752	
**Crystal size**	0.434 x 0.151 x 0.068 mm^3		0.4119 x 0.2174 x 0.1383 mm^3		0.457 x 0.031 x 0.023 mm^3	
**Theta range for data collection**	4.689 to 74.890°		3.071 to 29.587°		3.406 to 55.320°	
**Index ranges**	$-9 \le h \le 10, -22 \le k \le 19, -21 \le l \le 21$		$-10 \le h \le 9, -34 \le k \le 34, -25 \le l \le 20$		$-10 \le h \le 9, -13 \le k \le 13, -23 \le l \le 26$	
**Reflections collected**	11234		24502		15986	
**Independent reflections**	5332 [R(int) = 0.0220]		8758 [R(int) = 0.0406]		8238 [R(int) = 0.0552]	
**Completeness**	96.50% to theta = 67.684°		99.50% to theta = 25.242°		98.30% to theta = 55.320°	
**Absorption correction**	Gaussian		Gaussian		Gaussian	
**Max. and min. transmission**	1.00000 and 0.35593		1.00000 and 0.89047		1.00000 and 0.67209	
**Refinement method**	Full-matrix least-squares on F_2		Full-matrix least-squares on F_2		Full-matrix least-squares on F_2	
**Data / restraints / parameters**	5332 / 0 / 316		8758 / 0 / 466		8238 / 211 / 872	
**Goodness-of-fit on F^2**	1.469		1.148		1.037	
**Final R indices [I>2sigma(I)]**	R_1 = 0.0523, wR_2 = 0.1740		R_1 = 0.0423, wR_2 = 0.1226		R_1 = 0.1032, wR_2 = 0.2657	
**R indices (all data)**	R_1 = 0.0558, wR_2 = 0.1769		R_1 = 0.0607, wR_2 = 0.1561		R_1 = 0.1484, wR_2 = 0.3097	
**Largest diff. peak and hole**	1.020 and -1.079 e. $Å^{-3}$		0.975 and -1.249 e. $Å^{-3}$		2.182 and -2.577 e. $Å^{-3}$	
**Absolute structure parameter**	-		-		-	

Identification code	$H_{1.5}[Fe(L)_2](BF_4)_{3.5}\cdot 3CH_3NO_2$		$[Fe_nAg_{2n}(L)_{2n}(OH_2)_{2n}](BF_4)_{4n}$	
Empirical formula	$C_{45}H_{45}B_{3.5}F_{14}FeN_{21}O_6$		$C_{21}H_{18.5}AgB_{1.5}F_6Fe_{0.5}N_9O$	
Formula weight	1335.7		678.96	
Temperature	100.00(10) K		100(2) K	
Wavelength	1.54184 Å		1.54184 Å	
Crystal system	Triclinic		Orthorhombic	
Space group	$P\overline{1}$		*Cmca*	
Unit cell dimensions	a = 14.2058(4) Å	α= 90.798(2)°	a = 38.275(3) Å	α= 90°
	b = 15.3752(4) Å	β= 92.639(2)°	b = 22.727(2) Å	β= 90°
	c = 15.5408(4) Å	γ = 116.583(3)°	c = 15.2664(13) Å	γ = 90°
Volume	3030.06(16) $Å^3$		13279.8(18) $Å^3$	
Z	2		16	
Density (calculated)	1.464 mg/m^3		1.358 mg/m^3	
Absorption coefficient	2.947 mm^{-1}		7.110 mm^{-1}	
F(000)	1359		5392	
Crystal size	0.262 x 0.197 x 0.120 mm^3		0.347 x 0.080 x 0.057 mm^3	
Theta range for data collection	3.485 to 74.769°		4.525 to 39.960°	
Index ranges	-17 ≤ h ≤ 17, -19 ≤ k ≤ 19, -19 ≤ l ≤ 19		-31 ≤ h ≤ 31, -18 ≤ k ≤ 13, -11 ≤ l ≤ 12	
Reflections collected	51257		7578	
Independent reflections	12215 [R(int) = 0.0308]		2053 [R(int) = 0.0683]	
Completeness	100.00% to theta = 67.684°		99.10% to theta = 39.960°	
Absorption correction	Gaussian		Gaussian	
Max. and min. transmission	1.00000 and 0.70125		1.00000 and 0.50967	
Refinement method	Full-matrix least-squares on F2		Full-matrix least-squares on F2	
Data / restraints / parameters	12215 / 87 / 841		2053 / 1608 / 497	
Goodness-of-fit on F^2	1.609		1.239	
Final R indices [I>2sigma(I)]	R_1 = 0.1096, wR_2 = 0.3304		R_1 = 0.0922, wR_2 = 0.2520	
R indices (all data)	R_1 = 0.1140, wR_2 = 0.3411		R_1 = 0.1090, wR_2 = 0.2660	
Largest diff. peak and hole	3.466 and -0.783 e. $Å^{-3}$		1.667 and -0.612 e. $Å^{-3}$	
Absolute structure parameter	-		-	

3 References

[1] B. H. Dana, B. H. Robinson, J. Simpson, *J. Organomet. Chem.* **2002**, *648*, 251.
[2] O. CrysAlisPro; Agilent Technologies: Yarnton, England, 2012.
[3] L. J. Barbour, *J. Supramol. Chem.* **2001**, *1*, 189.
[4] G. Sheldrick, *Acta Cryst.* **2008**, *A64*, 112.
[5] L. Farrugia, *J. Appl. Cryst.* **1999**, *32*, 837.
